# An exacerbated phosphate starvation response triggers *Mycobacterium tuberculosis* glycerol utilization at acidic pH

**DOI:** 10.1128/mbio.02825-24

**Published:** 2024-11-29

**Authors:** Claire Healy, Sabine Ehrt, Alexandre Gouzy

**Affiliations:** 1 Department of Microbiology and Immunology, Weill Cornell Medical College, New York, New York, USA; St. Jude Children's Research Hospital, Memphis, Tennessee, USA

**Keywords:** *Mycobacterium tuberculosis*, inorganic phosphate, acid stress, glycerol, carbon metabolism

## Abstract

The mechanisms controlling *Mycobacterium tuberculosis* (Mtb) replication and survival inside its human host remain ill-defined. Phagosome acidification and nutrient deprivation are common mechanisms used by macrophages to restrict the replication of intracellular bacteria. Mtb stops replicating at mildly acidic pH (<pH5.8), an adaptive process called “acid growth arrest,” which is thought to play an important role in Mtb virulence. Using a genome-wide mutagenesis screen, we identified 95 genes whose disruption either decreases or increases Mtb fitness during acid growth arrest. We show that the virulence-associated inorganic phosphate (Pi) uptake system (Pst-1) regulates the ability of Mtb to replicate in acidic conditions. Deletion of *pstA1*, a gene coding for a subunit of the Pst-1 system, results in the overexpression of the Pi starvation regulator *regX3,* which is sufficient to restore Mtb growth in acid conditions. Our data further support the role of limited glycerol uptake and ROS-mediated glyceraldehyde-3-phosphate dehydrogenase (GAPDH) inhibition in causing Mtb acid growth arrest. This study reveals an unexpected role of the Pi starvation response in regulating acid growth arrest and highlights the intricacy of the mechanisms regulating redox homeostasis, acid stress response, and nutrient utilization in Mtb.

**IMPORTANCE:**

Despite the availability of antibiotic treatment, *M. tuberculosis* (Mtb), the causative agent of tuberculosis (TB), remains a major infectious disease killer worldwide. A better understanding of the environments that Mtb faces during infection and the mechanisms Mtb employs to respond and adapt may help identify currently unexplored pathways and targets for the development of novel anti-TB drugs. Here, we demonstrate that Mtb growth in acid can be restored by the over-expression of the Pi starvation response regulator *regX3*. This work paves the way toward a better understanding of the mechanisms controlling Mtb growth at acidic pH and highlights the role of inorganic phosphate in this process.

## INTRODUCTION

Tuberculosis (TB) remains a global leading cause of death due to a single infectious agent (1). After exposure to *Mycobacterium tuberculosis* (Mtb) via inhalation of bacilli into the lungs, the majority (~90%) of infections are asymptomatic in immunocompetent individuals. Although our immune system can control Mtb to prevent primary active disease, the bacteria are often not eradicated. Mtb bacilli can enter a non-replicating state and can remain dormant for years before resuming growth and causing active disease. Although antibiotic treatment is available, its long duration and associated toxicities as well as the emergence of drug resistance urge for the implementation of novel and more efficacious drug regimens.

The environments that bacteria face during infection can impact both bacterial replication and antibiotic efficacy (2–4). Therefore, a better understanding of the environments that Mtb faces during infection and the mechanisms employed by Mtb to respond and adapt may help identify currently unexplored pathways and targets for the development of novel anti-TB drugs. Mtb bacilli are found inside lung macrophages, which represent both the main site of bacterial growth and containment (5–7). The replication status of Mtb during infection is tightly linked to the status of the host’s immune system (8–10). Immune cells produce antimicrobial compounds, such as hydrogen peroxide and nitric oxide, to directly kill Mtb. Moreover, immune cells also reshape the local environment to limit Mtb growth by, for example, restricting essential nutrient availability (e.g., iron), reducing access to oxygen, and increasing the local acidity (11, 12). Mtb has developed mechanisms to sense and quickly adapt to those physicochemical changes, notably by the utilization of two-component systems (13, 14).

Mtb replication is inhibited at mildly acidic pH (pH <5.8) in standard culture media containing the glycolytic carbon sources glycerol and glucose. This non-replicating state also known as “acid growth arrest” is proposed to be an adaptive process playing a role in Mtb virulence and antibiotic tolerance (15, 16). However, Mtb can grow at acidic pH in the presence of alternative carbon sources such as pyruvate and lipids (e.g., oleic acid, cholesterol), revealing that the impact of acidic pH on Mtb growth depends on the nature of the available carbon source (17, 18). How the interplay between pH and central carbon metabolism controls Mtb replication remains elusive.

We previously demonstrated that in Mtb, glycerol assimilation into central carbon metabolism is reduced at acidic pH, and this correlates with a decrease in the activity of glyceraldehyde-3-phosphate dehydrogenase (GAPDH), an enzyme necessary for the incorporation of glycerol-derived carbon into the tricarboxylic acid cycle (17). Other mechanisms, such as nutrient uptake, also seem to play an important role in Mtb acid growth arrest. Indeed, gain of function mutations in the gene *ppe51*, coding for a glycerol import system, enhanced glycerol uptake and alleviated Mtb growth inhibition at acidic pH (16, 19). In this study, we employed a genome-wide mutagenesis screen to identify the molecular mechanisms altering Mtb acid growth arrest and identified an inorganic phosphate uptake system that plays an important role in orchestrating this process.

## RESULTS

### Identification of genes altering Mtb fitness at acidic pH with glycerol and glucose as main carbon sources

**Fig 1:**
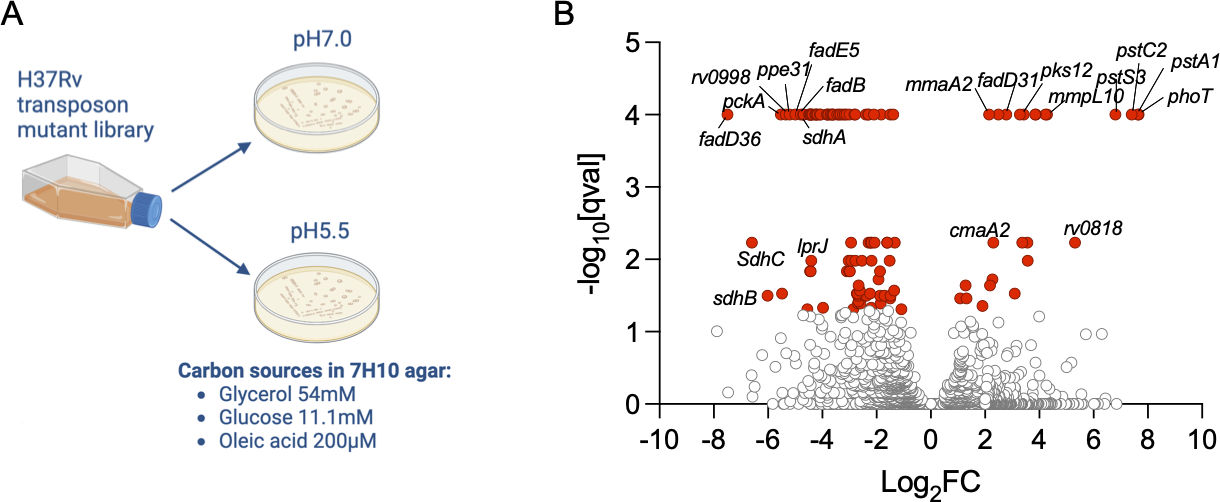
Identification of genes altering Mtb fitness in acidic conditions in the presence of glycerol and glucose as main carbon sources. (**A**) Schematic of experimental design to identify transposon mutants with altered ability to grow or survive on solid acidified media. (**B**) Volcano plot displaying genes where transposon insertion mutations resulted in significantly altered representation (red dots) within the transposon mutant library at pH 5.5 compared with pH 7. For each gene, the ratio of normalized sequence reads per insertion site (pH 5.5 /pH 7) is plotted on the *X* axis (log_2_ fold change). The *Y* axis represents the significance of each of these changes in representation (q value). Data are from three biological replicate experiments.

Mtb displays a 7–10-day long growth lag when cultured at pH 5.5 in standard media (Middlebrook 7H9) containing glycerol and glucose as main carbon sources (Fig. S1). We used transposon mutagenesis coupled with next-generation sequencing (Tn-seq) to identify the genetic determinants involved in this acid growth arrest. We cultured a saturated transposon mutant library (20) on solid media (Middlebrook 7H10) buffered to either pH 7 or pH 5.5 (Fig. 1A). Transposon mutants were harvested after 3 weeks (pH 7) or 6 weeks (pH 5.5) of incubation at 37°C. Mutants with transposon insertions in 95 genes were shown to be significantly underrepresented (*n* = 74) or overrepresented (*n* = 21) at pH 5.5 compared with pH 7 (Fig. 1B; File S1).

### Underrepresented mutants at acidic pH

Among the genes whose disruption decreased Mtb fitness at acidic pH were genes related to carbon metabolism such as beta-oxidation (*rv1193*/*fadD36*, *rv0244c*/*fadE5, rv0859/fadA,* and *rv0860*/*fadB*) and gluconeogenesis (*rv0211*/*pckA*). Interruption of *pckA* leading to decreased Mtb fitness at acidic pH is consistent with the growth defect observed for a Δ*pckA* mutant at mildly acidic pH (pH 5.7) with glycerol as the only carbon source (16). The requirement for gluconeogenesis for Mtb growth, even in a medium where lipids are not provided as the main carbon source, is attributed to the importance of anaplerosis in Mtb during adaptation to acidic pH (16).

We also identified transposon insertions underrepresented in acidic conditions in genes encoding for enzymes whose function bridges both carbon metabolism and respiration such as those encoding the subunits of succinate dehydrogenase (SDH) complex 1 (*rv0247c, rv0248c,* & *rv0249c*) and complex 2 (*rv3316* & *rv3318*) as well as *rv0998,* which encodes the lysine acetyltransferase protein PAT (21, 22). We confirmed the growth defects on acidified agar plates of deletion mutants of *pckA* and the SDH complex 1 encoding genes (Fig. S2A).

### Overrepresented mutants at acidic pH

**Fig 2:**
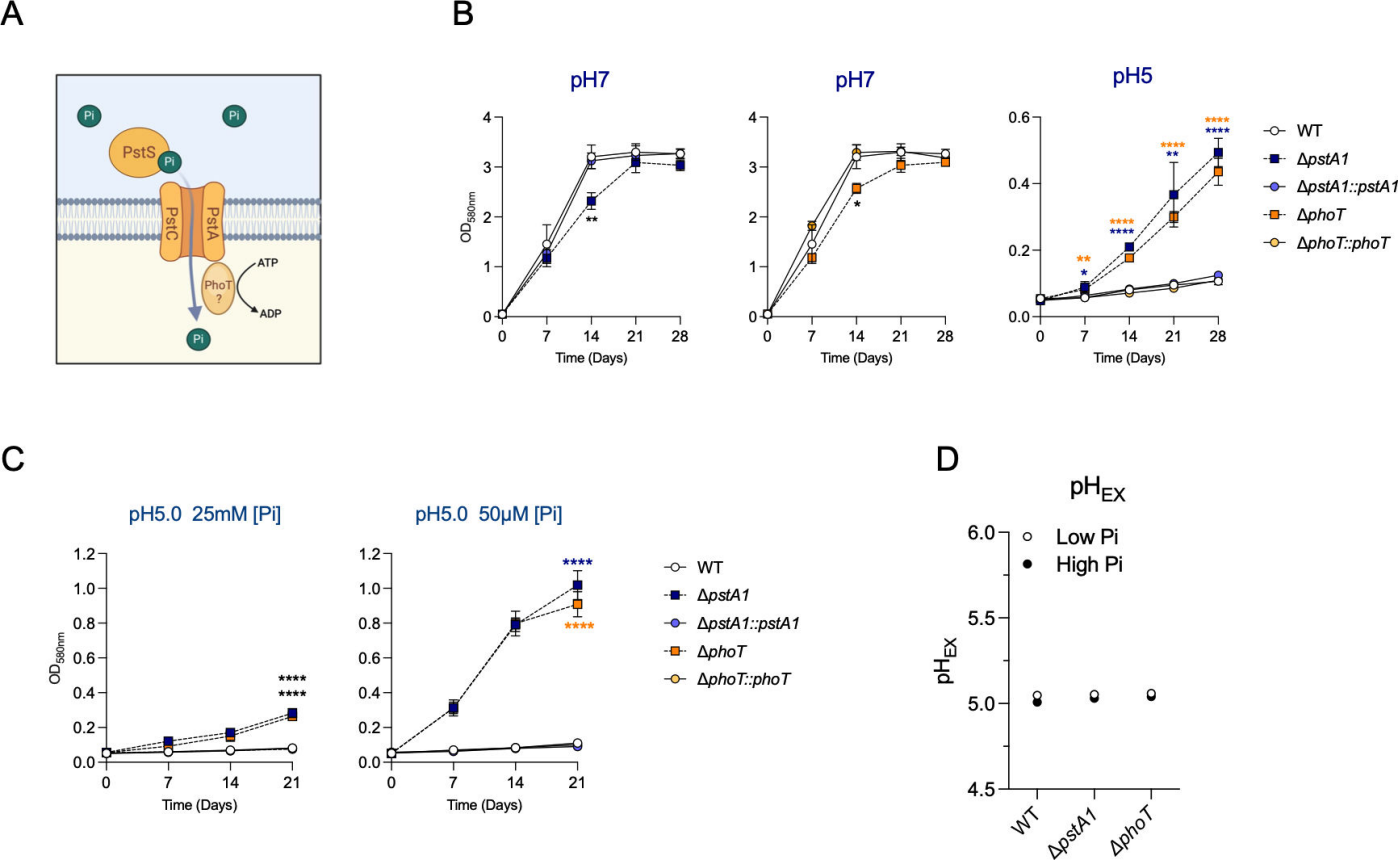
Deletion of the phosphate transport-related genes *pstA1* and *phoT* permits Mtb growth at acidic pH. (**A**) Proposed schematic of Pst-1 phosphate uptake system. (**B**) Growth curves of wild-type Mtb (WT), knock-out strains for *pstA1* (Δ*pstA1*) and *phoT* (Δ*phoT*), and complemented mutants (Δ*pstA1::pstA1*, Δ*phoT::phoT*) in 7H9-0.2%glycerol-10%ADN media with pH adjusted to 7 or 5. (**C**) Growth of WT, Δ*pstA1,* Δ*pstA1::pstA1,* Δ*phoT,* and Δ*phoT::phoT* in 7H9-0.2%glycerol-10%ADN media with high inorganic phosphate (Pi) (25 mM) or low Pi (50 µM) conditions at pH 5. (**D**) Extracellular pH (pH_EX_) of cultures from (**C, D21**). Growth was monitored by measurement of optical density (OD_590nm_). Data from B-D are the means and standard deviations of three independent experiments. Statistical significance was determined by ordinary one-way ANOVA. * = adj *P*-value <0.05, ** = adj *P*-value <0.005, **** = adj *P*-value <0.0001.

Strikingly, we identified four genes related to phosphate transport whose interruption increased Mtb fitness in acidic conditions (Fig. 1B; File S1). Three of these (*rv0928/pstS3*, *rv0929/pstC2, rv0930*/*pstA1*) belong to an operon encoding an ABC transport system dedicated to the uptake of inorganic phosphate (Pi) with high affinity and known as Phosphate-Specific Transport (Pst) that we will refer to as Pst-1 (23) (Fig. S3A). The fourth gene (*rv0820/phoT*) is located elsewhere in the genome and encodes a putative phosphate transport ATP-binding protein. Pst transporters are composed of four subunits: a periplasmic phosphate-binding protein (PstS), two membrane-spanning components (PstA and PstC), and an ATP-binding protein (PstB) that binds to and hydrolyzes ATP to provide the energy necessary for Pi transport (Fig. 2A). The Pst-1 locus lacks a PstB component necessary to bind and hydrolyze ATP. Because PhoT is predicted to function as a PstB protein, and transposon insertion mutations in the *phoT* gene led to a similar fitness benefit as disruptions of the three Pst-1 genes at acidic pH, we hypothesize that PhoT interacts with Pst-1 subunits to form a functional Pst-1 transporter. The Mtb genome contains three other systems predicted to allow Pi import. Another complete Pst system that we will refer to as Pst-2 is encoded by *rv0933*/*pstB; rv0934/pstS1; rv0935/pstC1; and rv0936/pstA2* and predicted to import Pi with high affinity, and two low-affinity Pi permeases encoded by *rv0545c*/*pitA* and *rv2281*/*pitB* (Fig. S3A). Only disruption of *pst-1* and *phoT* genes led to a fitness benefit in acidic conditions (Fig. S3B).

### Deletion of *pstA1* and *phoT* enables Mtb to grow at acidic pH with glycerol and glucose as main carbon sources

We constructed deletion mutants of *pstA1* (Δ*pstA1*) and *phoT* (Δ*phoT*) (see Supplementary Material and Methods) and confirmed their growth advantage in comparison to WT Mtb on acidified agar plates (pH 5.5) (Fig. S2B). As we and others have shown that Mtb acid growth arrest occurs at pH <5.8 (17, 18, 24), pH 5 was used for the remainder of this study to ensure Mtb growth restriction and facilitate the detection of any enhanced acid growth phenotype. At pH 7, despite a slight growth defect of Δ*pstA1* and Δ*phoT* at an early stage of growth, all strains reached similar biomass after 28 days (Fig. 2B). Consistent with our Tn-seq screen, both Δ*pstA1* and Δ*phoT* grew in acidified 7H9 medium (pH 5) containing glycerol and glucose as main carbon sources, whereas the WT parental strain and complemented strains failed to grow (Fig. 2B).

We next investigated the impact of Pi availability on growth of the mutants at acidic pH.

Pi is essential for bacterial growth and as expected, at Pi concentrations below 195 µM, growth of all strains was reduced at pH 7, indicating insufficient phosphate amounts to support robust bacterial growth (Fig. S4A). In acidic conditions, WT and complemented mutants did not grow at any of the Pi concentrations tested. In contrast, both Δ*pstA1* and Δ*phoT* replicated in acidic conditions. Strikingly, reducing Pi concentrations resulted in increasingly robust growth of the mutants at acidic pH (Fig. S4B).

Growth of Δ*pstA1* and Δ*phoT* was indeed enhanced in low Pi (50 µM) versus high Pi (25 mM) media at pH 5 (Fig. 2C). These data imply that PstA1 and PhoT are not necessary for Pi uptake in Mtb. The second phosphate-specific transporter Pst-2 and the permeases PitA and PitB (Fig. S3A), which are predicted to allow Pi uptake with high and low affinity, respectively, most likely compensate for the loss of PstA1 and PhoT functions. In accordance, *pitB* gene expression is increased in a Δ*pstA1* mutant (25). We confirmed the growth advantage of Δ*pstA1* and Δ*phoT* was not due to an ability to neutralize the acidity of the medium as the culture media remained acidic throughout the experiment (Fig. 2D). The enhanced growth of Δ*pstA1* in acidic conditions contrasts with the acid stress sensitivity of Δ*pstA1* previously reported (26). This previous study used the detergent Tween 80, which becomes toxic to Mtb at acidic pH (27). We confirmed that Tween 80 is responsible for the previously reported survival defect of Δ*pstA1* at acidic pH (Fig. S5).

### Deletion of *pstA1* and *phoT* enables Mtb to use glycerol but not glucose as a main carbon source at acidic pH

**Fig 3:**
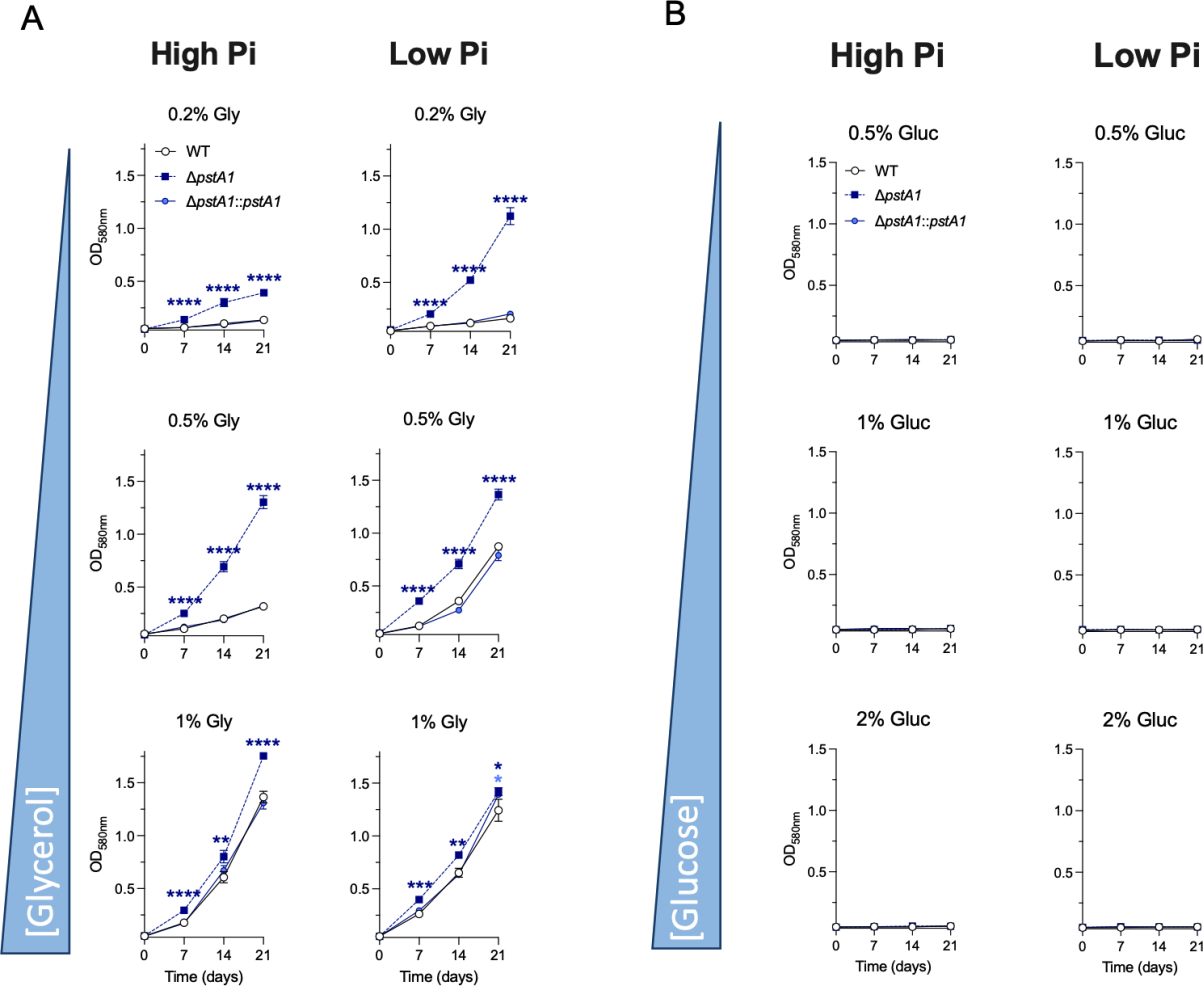
Mtb lacking *pstA1* displays an increased ability to utilize glycerol but not glucose to grow at acidic pH. Growth of wild-type Mtb (WT), Δ*pstA1,* and complemented mutant (Δ*pstA1*::Δ*pstA1*) in 7H9 media at pH 5 with high inorganic phosphate (Pi) (25 mM) or low Pi (50 µM). Growth was determined for each strain in a gradient of glycerol (Gly) (**A**) or glucose (Gluc) (**B**) concentrations (% vol/vol) that serve as main carbon source. Growth was monitored by measurement of optical density (OD_590nm_). Data are the means and standard deviations of three independent experiments. Statistical significance was determined by ordinary one-way ANOVA with Tukey multiple comparisons test. **:*P* value ≤0.01, ****: *P*-value <0.0001.

We examined the growth of Δ*pstA1* and Δ*phoT* in media containing either glycerol or glucose as the main carbon source to determine whether their growth advantage at acidic pH was due to a restored utilization of either carbon source. Both mutants displayed a growth advantage in acidic conditions with 0.2% glycerol (Fig. 3A; Fig. S6A). We then investigated whether reduced glycerol utilization in WT Mtb could be overcome by providing increased amounts of glycerol. Indeed, the growth inhibition of WT Mtb at acidic pH was relieved by increasing the amount of glycerol to 0.5%, and this was enhanced in low Pi conditions. The Pi-dependent effect became negligible with further increased amounts of glycerol (1%). On the contrary, glucose, even at high concentrations (~ 100 mM), did not support Mtb growth at acidic pH (Fig. 3B; Fig. S6B).

### RegX3 overexpression is required for Mtb growth at acidic pH with glycerol as the main carbon source

**Fig 4:**
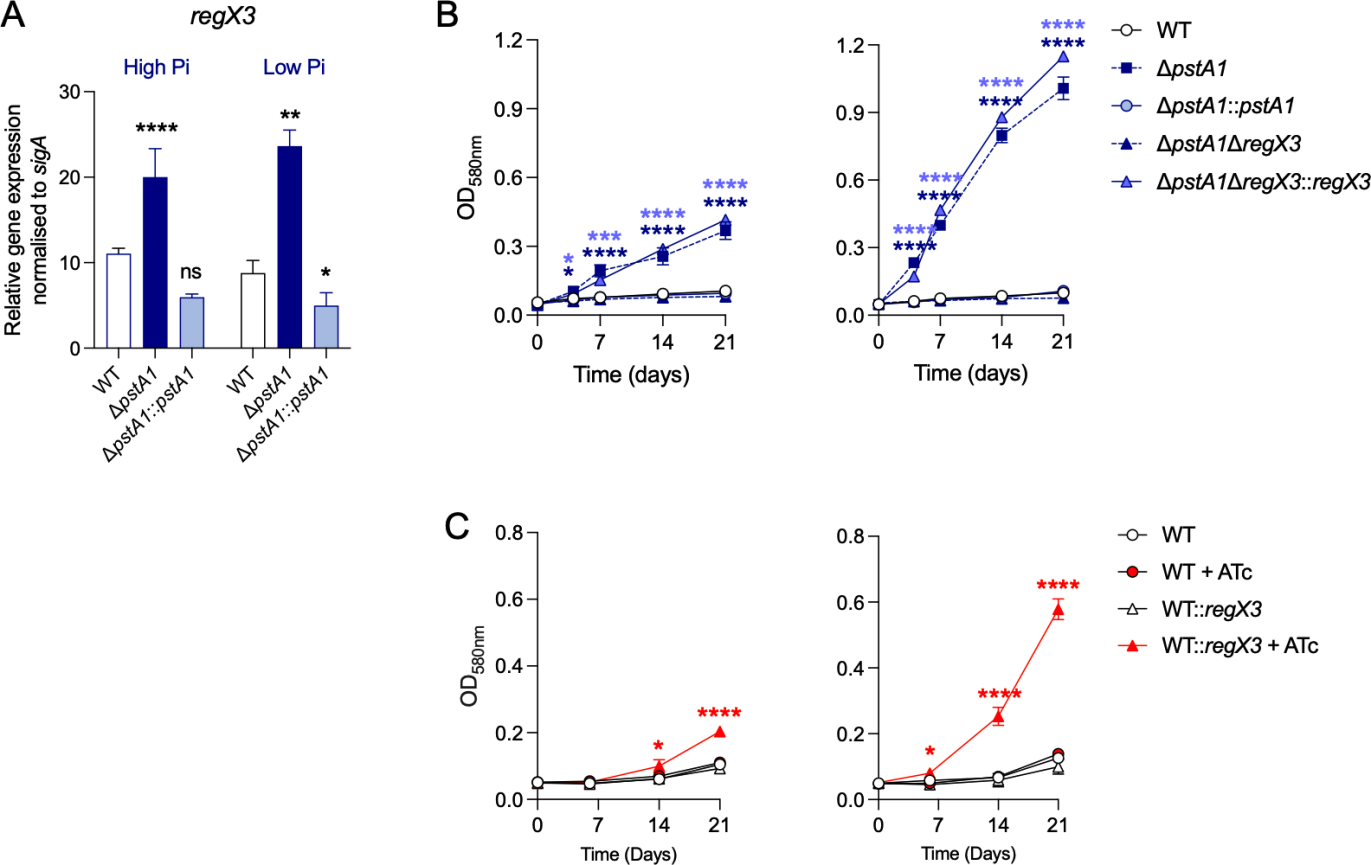
RegX3 overexpression permits Mtb growth at pH 5 with glycerol as the main carbon source. (**A**) Relative gene expression of *regX3* in wild-type Mtb (WT), knock-out strain for *pstA1* (Δ*pstA1*), and complemented mutant (Δ*pstA1::pstA1*) cultured in 7H9-0.2%glycerol media at pH 5 with high Pi (25 mM) or low Pi (50 µM). (**B**) Growth curves of WT, Δ*pstA1*, Δ*pstA1::pstA1*, double knock-out Δ*pstA1*Δ*regX3,* and complemented double knock-out (Δ*pstA1*Δ*regX3::regX3*) in 7H9-0.2%glycerol media at pH 5 with high inorganic phosphate (Pi) (25 mM) (left panel) or low Pi (50 µM) (right panel). (**C**) Growth curves of WT Mtb and WT containing a second copy of *regX3* under the control of an ATc inducible promoter (WT::*regX3*) cultured in 7H9-0.2%glycerol media at pH 5 with high (Hpi; 25 mM) (left panel) or low (Lpi; 50 µM) Pi (right panel). Anhydrotetracycline ([ATc]= 500 ng/mL) was added to cultures to induce *regX3* expression. Growth was monitored by measurement of optical density (OD_590nm_). Data are the means and standard deviations of three independent experiments. Statistical significance was determined by ordinary one-way ANOVA with Tukey multiple comparison test. * *P*-value ≤0.05, ** *P* value ≤0.01, *** *P*-value ≤0.001, **** *P*-value <0.0001. Two-way ANOVA with Sidak’s multiple comparisons test on data in A found no significant difference in *regX3* expression in the strains when comparing High Pi to Low Pi.

In bacteria, Pi utilization is often regulated by a Pst system and an associated two-component system (TCS) that controls the expression of genes involved in Pi uptake (28, 29). In Mtb, the TCS responding to Pi starvation response is SenX3/RegX3 (30, 31), and the loss of *pstA1* function results in increased expression of the transcriptional regulator RegX3 (25). We first confirmed that *regX3* was overexpressed in *ΔpstA1* compared with WT at acidic pH (Fig. 4A). To determine whether RegX3 plays a role in the phosphate-regulated growth of Δ*pstA1* at acidic pH, we then examined the growth of Mtb lacking both the *pstA1* and *regX3* genes (Δ*pstA1*Δ*regX3*) at acidic pH with glycerol as the main carbon source. Δ*pstA1*Δ*regX3* failed to grow at acidic pH, regardless of Pi concentrations. Expression of the *regX3* gene in Δ*pstA1*-Δ*regX3* (Δ*pstA1*-Δ*regX3::regX3*) restored growth similar to that of Δ*pstA1* (Fig. 4B). This indicates that Δ*pstA1* requires RegX3 to utilize glycerol to grow at acidic pH.

To test whether RegX3 overexpression, outside of the context of a disrupted Pst-1 system, facilitates utilization of glycerol at acidic pH, we expressed a second copy of *regX3* in WT Mtb (WT::*regX3*) under the control of an anhydrotetracycline (ATc) inducible promoter. Inducing expression of this second *regX3* copy was sufficient to promote Mtb growth at pH 5 in the presence of glycerol and was enhanced in low Pi media similarly to what is observed for *ΔpstA1* and *ΔphoT* mutants (Fig. 4C; Fig. S6A and S7). These data show that an exaggerated Pi starvation response, via *regX3* overexpression, is driving the acid growth phenotype of Δ*pstA1*.

### PE19 facilitates growth of Δ*pstA1* at acidic pH with glycerol as the main carbon source

**Fig 5:**
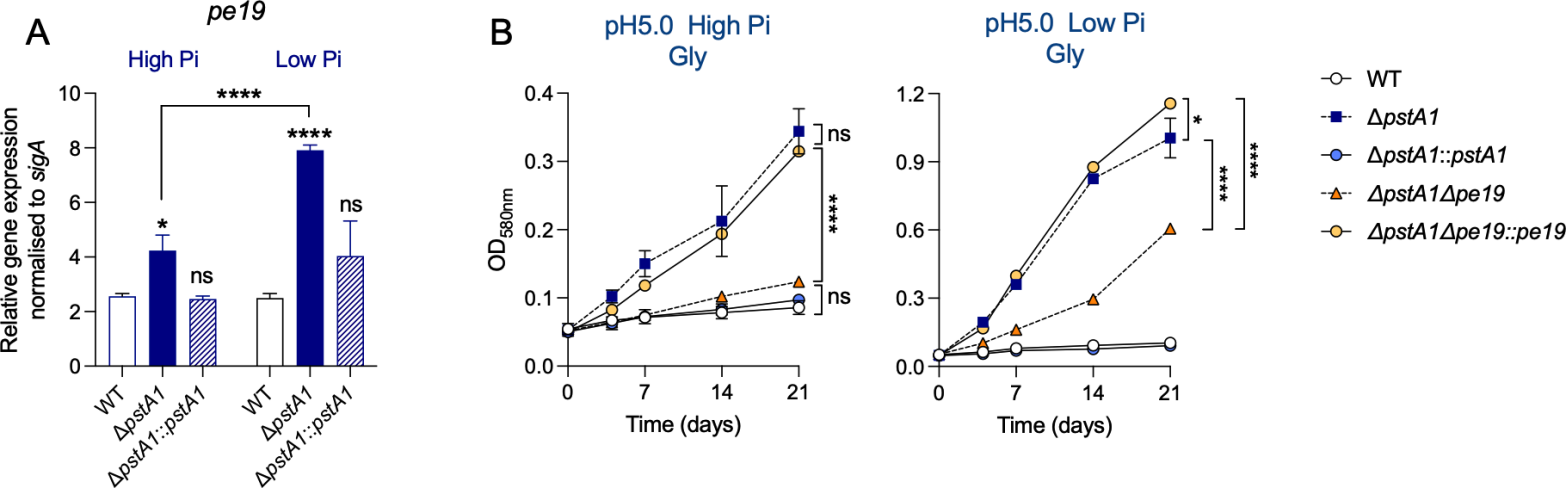
PE19 is required for optimal utilization of glycerol by Δ*pstA1* at acidic pH. (**A**) Gene expression analysis of *pe19* in wild-type (WT), *pstA1* knock-out (Δ*pstA1*), and complemented mutant (Δ*pstA1::pstA1*) in 7H9-0.2% glycerol at pH 5 with high inorganic phosphate (Pi) (25 mM) or low Pi (50 µM). (**B**) Growth curve experiments of WT, Δ*pstA1,* Δ*pstA1::pstA1,* the double knock-out (Δ*pstA1Δpe19*), and complemented double knock-out (Δ*pstA1*Δ*pe19::pe19*) in 7H9-0.2% glycerol media at pH 5 with high or low Pi. Growth was monitored by measurement of optical density (OD_590nm_). Data are the means and standard deviations of three independent experiments. Statistical significance was determined by one-way ANOVA and Tukey multiple comparisons test * *P*-value ≤0.05, **** *P*-value <0.0001.

The PE/PPE gene family is the most highly expressed during Mtb Pi starvation (30). PE/PPE proteins are involved in various biological processes including nutrient uptake. Induced expression of *pe*/*ppe* genes in response to Pi-limiting conditions could therefore be a mechanism to potentiate Pi uptake. Several *pe*/*ppe* genes were found to be overexpressed in Δ*pstA1*, even in a Pi-rich environment, due to the constitutive overexpression of *regX3* (25, 26). PE19 was previously shown to be responsible for the increased permeability of Δ*pstA1* (26). We next investigated whether the RegX3-dependent overexpression of *pe19* in Δ*pstA1* was responsible for its ability to utilize glycerol to grow at acidic pH. First, we confirmed that *pe19* gene was indeed over-expressed in Δ*pstA1* in all conditions tested (Fig. 5A; Fig. S8).

Deletion of *pe19* in Δ*pstA1* (Δ*pstA1*Δ*pe19*) prevented growth at acidic pH in phosphate-replete media and the double mutant displayed a substantial growth defect in low Pi conditions relative to Δ*pstA1* (Fig. 5B). This demonstrates that PE19 is required for optimal growth of Δ*pstA1* on glycerol at acidic pH. Moreover, Δ*pstA1*Δ*pe19* grew similarly to WT at acidic pH independent of Pi concentration in the presence of oleic acid (Fig. S9). This suggests that PE19 is required specifically for the utilization of glycerol by Δ*pstA1*, most likely by promoting an increase in glycerol uptake at acidic pH. Supporting this, mutants with transposon insertions in *pe19* were significantly underrepresented in our screen at pH 5.5 (File S1).

Altogether, our data reveal that Δ*pstA1* relies partly on PE19 to utilize glycerol for growth in acidic conditions. Nevertheless, the deletion of *pe19* did not fully prevent growth of Δ*pstA1* at acidic pH in low Pi conditions, suggesting that other RegX3-dependent mechanism(s) might contribute to the ability of Δ*pstA1* to utilize glycerol at acidic pH.

### Δ*pstA1* retains glyceraldehyde-3-phosphate dehydrogenase enzymatic activity at acidic pH in a RegX3-dependent manner

**Fig 6:**
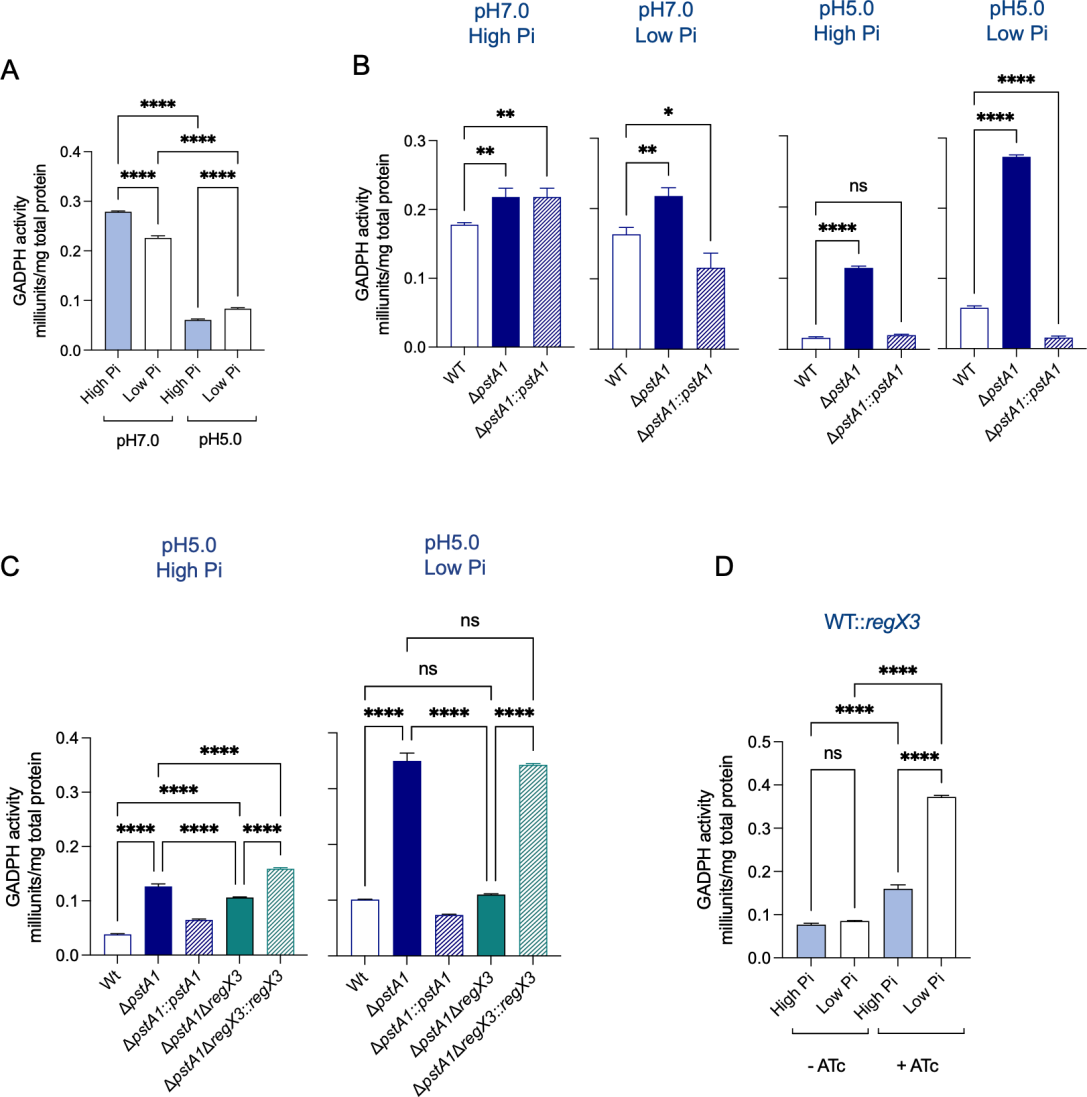
Increased GAPDH activity at acidic pH in Mtb lacking *pstA1 is* dependent on phosphate levels and RegX3 expression. (**A**) GAPDH activity in cell lysates from wild-type (WT) Mtb cultured in 7H9-0.2% glycerol media at pH 7 or pH 5 with high inorganic phosphate (Pi) (25 mM) or low Pi (50 µM). (**B**) GAPDH activity in cell lysates from WT, *pstA1* knock-out mutant (Δ*pstA1*), and complemented mutant (Δ*pstA1::pstA1*) cultured in 7H9-0.2% glycerol at pH 7 or pH 5 in Hpi or Lpi. (**C**) GAPDH activity in cell lysates from WT, Δ*pstA1,* Δ*pstA1::pstA1,* Δ*pstA1ΔregX3,* and Δ*pstA1ΔregX3::regX3* cultured in 7H9-0.2% glycerol media at pH 5 with high Pi or low Pi. (**D**) GAPDH activity in Mtb with inducible overexpression of *regX3* (WT::*regX3*) by addition of anhydrotetracycline ([ATc]= 500 ng/mL). (**A-D**) Lysates were collected after 5 days of incubation of the bacteria in the tested media. Representative of at least three independent experiments. Statistical significance was determined by one-way ANOVA with Tukey multiple comparisons test * *P*-value ≤0.05, **P value ≤0.01, **** *P*-value <0.0001.

The activity of GAPDH is reduced in Mtb exposed to acidic conditions, which is associated with a reduction in glycolysis flux and glycerol assimilation into the TCA cycle leading to growth arrest (17). Considering the enhanced capacity of Δ*pstA1* to grow at acidic pH in the presence of glycerol, we measured GAPDH activity in Δ*pstA1*. GAPDH activity was reduced in WT Mtb in response to acid stress, as previously shown, and only slightly affected by Pi (Fig. 6A). However, GAPDH activity in Δ*pstA1* was substantially higher than in WT at acidic pH (Fig. 6B). Strikingly, GAPDH activity in Δ*pstA1* was not reduced in acidic and low Pi conditions. In accordance with this, we found that the enhanced GAPDH activity in Δ*pstA1* depends on RegX3 as Δ*pstA1*-Δ*regX3* had reduced GAPDH activity in comparison to Δ*pstA1,* and this was complemented by the expression of *regX3* in Δ*pstA1*Δ*regX3* (Δ*pstA1*-Δ*regX3::regX3*) (Fig. 6C). Furthermore, ATc-induced expression of a second copy of *regX3* in WT (WT::*regX3*) resulted in increased GAPDH activity at acidic pH specifically in low Pi conditions (Fig. 6D). Altogether, our data reveal that the RegX3-mediated Pi starvation response can restore GAPDH activity at acidic pH.

### Lack of *pstA1* in Mtb decreases the level of acid-induced reactive oxygen species (ROS) in a RegX3-dependent manner

**Fig 7:**
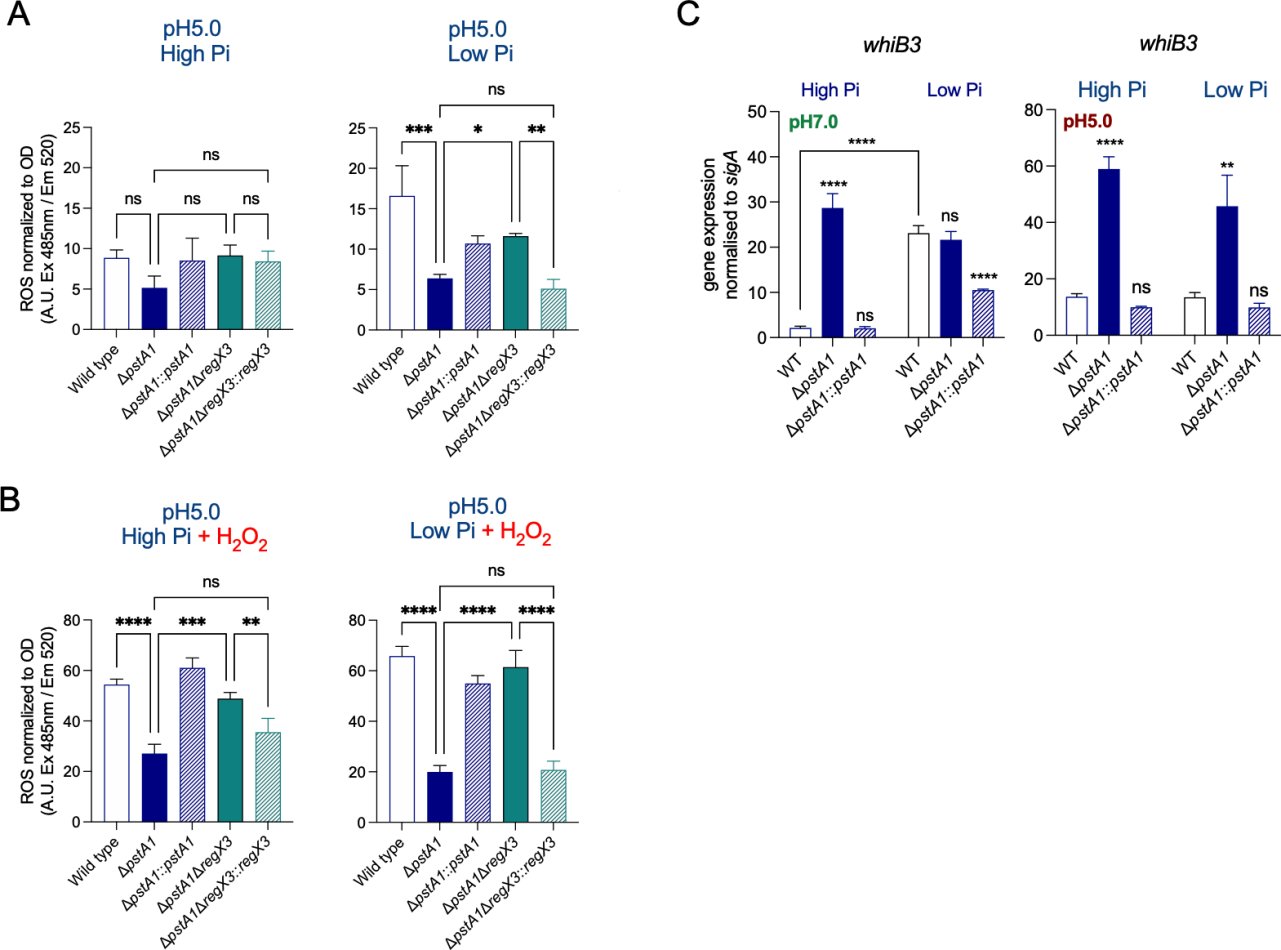
Mtb lacking functional Pst-1 maintains lower ROS levels under conditions of limiting phosphate and oxidative stress in acidic conditions. (**A**) Measurements of intracellular ROS in Mtb strains cultured for 5 days in 7H9-0.2% glycerol at pH 5 with high (25 mM) or low (25 µM) inorganic phosphate (Pi). (**B**) Same experiment as in (**A**) with the addition of hydrogen peroxide H_2_O_2_ (1 mM) to the cultures to induce oxidative stress during ROS measurements. Levels of ROS in (**A**) and (**B**) were measured by fluorescence measurement of the Cellrox Green probe (5 µM) 2 h after its addition. (**C**) Gene expression analysis of *whiB3* in wild type (WT), *pstA1* knock-out mutant (Δ*pstA1*), and complemented mutant (Δ*pstA1::pstA1*) in 7H9-0.2%glycerol at pH 5 or pH7 with high (25 mM) or low (50 µM) Pi. Data in A and B are the means and standard deviations and representative of two experiments done in triplicates. Data in C are the means and standard deviations of three biological replicates. Statistical significance was determined by ordinary one-way ANOVA with Tukey multiple comparisons test. * *P*-value ≤0.05, ** *P* value ≤0.01, *** *P*-value ≤0.001, **** *P*-value <0.0001.

The gene encoding GAPDH (*gap*/*rv1436*) is not predicted to belong to the *regX3* regulon as the expression level of *gap* is not affected by the deletion of either *regX3* (abolishing RegX3-dependent regulation) or *pstA1* (causing overexpression of *regX3*) (25, 31). Accordingly, GAPDH protein levels were similar in WT and Δ*pstA1* in all growth conditions tested (Fig. S10). This suggests that GAPDH activity in Δ*pstA1* is post-translationally regulated. GAPDH activity is affected by reactive oxygen species (ROS) in eukaryotes and bacteria due to oxidation of its active site cysteine causing inhibition of its activity (17, 32–35). In Mtb, reduced GAPDH activity correlated with the production of endogenous ROS in response to acid stress (acid-induced ROS) (17, 36). The amount of acid-induced ROS was lower in Δ*pstA1* than in WT (Fig. 7A) and associated with increased GAPDH activity suggesting that GAPDH is less prone to oxidation-dependent inhibition in Δ*pstA1*. Moreover, addition of exogenous hydrogen peroxide (H_2_O_2_) increased ROS in bacteria facing acid stress and demonstrated the capacity of Δ*pstA1* to maintain low levels of ROS (Fig. 7B). The capacity of Δ*pstA1* to limit ROS was dependent on RegX3, especially at low Pi conditions, which suggests a role of RegX3 in ROS detoxification (Fig. 7A and B). Finally, to examine the role of RegX3 in ROS detoxification further, we measured acid-induced ROS levels in Mtb either lacking (Δ*regX3*) or overexpressing *regX3* (WT::*regX3* + ATc) and observed no significant change in ROS levels in bacteria lacking RegX3 relative to WT. However, overexpression of *regX3* in WT (WT::*regX3* + ATc) resulted in lower ROS levels in agreement with the enhanced capacity of this strain to utilize glycerol to grow at acidic pH (Fig. S11; Fig. 4C).

### Lack of *pstA1* in Mtb increases the expression of the redox-sensing transcription factor WhiB3

In addition to regulating the response of Mtb to Pi-limiting conditions, the two-component system SenX3/RegX3 is also described as a redox regulating system (37). The expression of a cytosolic redox sensor WhiB3 is directly induced by RegX3 in response to low Pi and acidic conditions, separately (38, 39). Moreover, *whiB3* is among the top 10 genes overexpressed in Δ*pstA1* compared with WT in standard growth conditions (25).

Knowing the key role of WhiB3 in mitigating oxidative stress in Mtb, we examined *whiB3* expression in Δ*pstA1* in response to pH and Pi levels. *whiB3* expression was increased in WT Mtb in response to either acidic pH (pH 7 vs pH 5 in high Pi conditions) or low Pi conditions (high Pi vs low Pi at pH 7) as previously reported (39) (Fig. 7C). Expression of *whiB3* was also increased in Δ*pstA1* compared with WT at neutral pH and high Pi. WT and Δ*pstA1* displayed a similar abundance of *whiB3* transcripts at neutral pH and low Pi likely due to the increase in *whiB3* expression in WT in response to low Pi levels (Fig. 7C).

Strikingly, the increased *whiB3* expression in Δ*pstA1* was further enhanced by acidic pH (Fig. 7C). Given the important role of WhiB3 in counteracting oxidative stress in Mtb, this suggests that *whiB3* may play a role in limiting ROS in Δ*pstA1*. Other ROS detoxifying systems that could reduce ROS levels such as the alkyl hydroperoxidase AhpC/D shown previously to be overexpressed in Δ*pstA1* (25, 40) or the cytochrome *bd* oxidase, which is directly regulated by RegX3 (41, 42), did not show significant changes in expression in Δ*pstA1* compared to WT in response to reduced pH or Pi levels (Fig. S12).

## DISCUSSION

**Fig 8:**
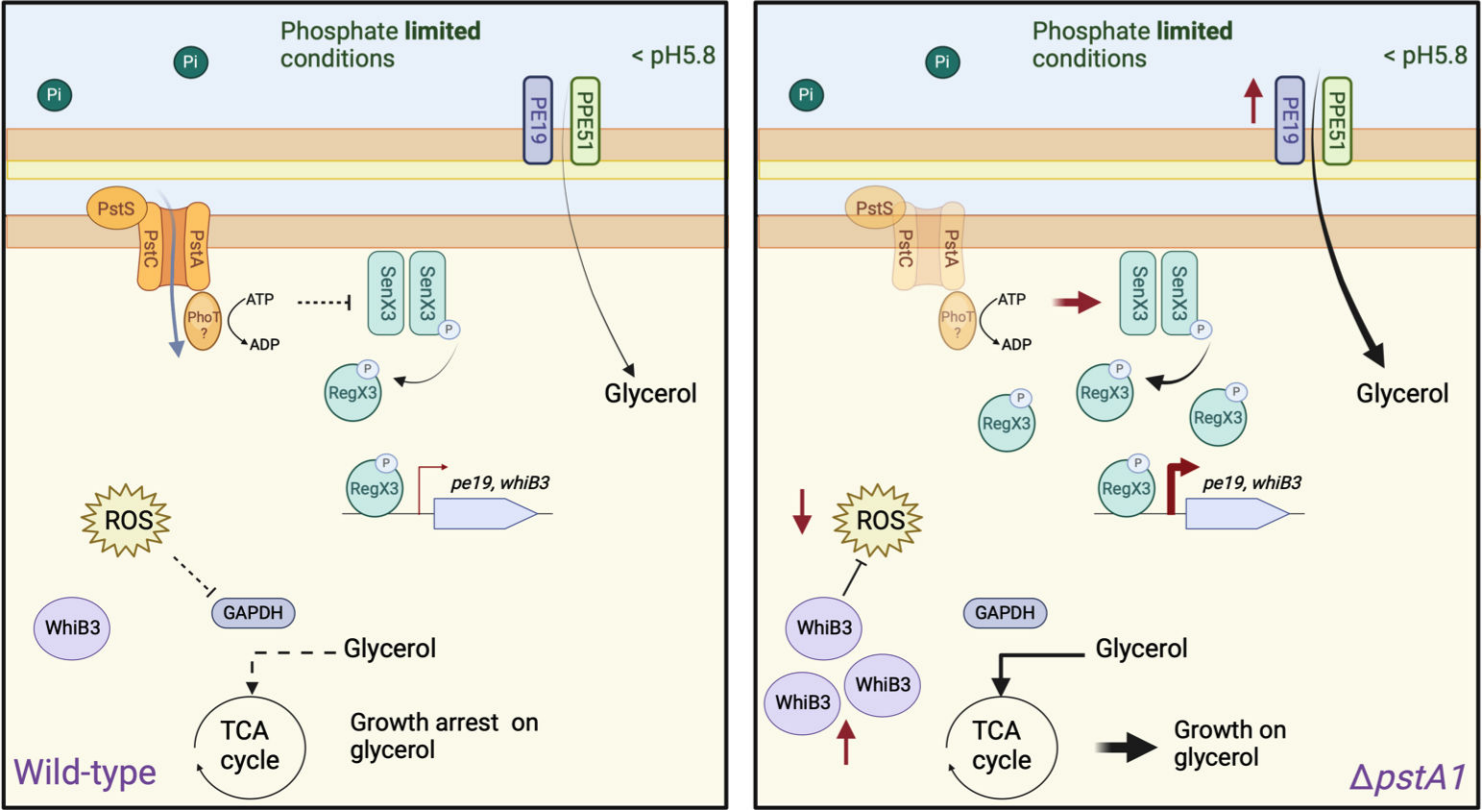
Schematic of our model comparing the response of wild-type and ∆*pstA1* Mtb strains under acidic and phosphate limiting conditions. Under phosphate (Pi) limited and acidic conditions (< pH 5.8), wild-type Mtb is unable to grow using glycerol (0.2%) resulting from a reduction in glycerol uptake and assimilation due to the inhibition of the glyceraldehyde-3-phosphate dehydrogenase (GAPDH) enzyme activity by acid-induced ROS (left panel). A lack of Pst-1 function due to the deletion of the genes *pstA1* or *phoT* causes an increase in the activity of the two-component system SenX3/RegX3 causing an induction in *regX3* gene expression (and probably RegX3 activation by phosphorylation from its cognate kinase SenX3). Higher RegX3 amounts increase the expression of RegX3 regulated genes such as *pe19* and *whiB3*. The higher PE19 amounts might increase the formation of the porin-like PE19-PPE51 and enhance glycerol uptake. In addition, the increased expression of the redox-sensing transcription factor gene *whiB3* might help reduce the amount of acid-induced ROS and preserve GAPDH activity. This RegX3-dependent exacerbated Pi starvation response allows Mtb to utilize glycerol as a main carbon source to grow in acidic conditions (<pH 5.8).

Pst systems are known to inhibit signal transduction from Pi-specific TCS in phosphate-replete conditions. In *Escherichia coli* and *Salmonella*, Pst inhibits the TCS PhoR/PhoB in phosphate-replete conditions, and a lack of Pst activity was shown to lead to the overactivation of PhoR/PhoB (43, 44). Similarly in Mtb, loss of Pst-1 function due to deletion of *pstA1* leads to the overexpression of *regX3,* and most phenotypes observed in Δ*pstA1* were shown to be abrogated upon deletion of *regX3* (25). In accordance with this, we observed that the capacity of Δ*pstA1* to use glycerol to support growth at acidic pH depends on RegX3. Moreover, overexpression of *regX3* is sufficient to prevent the entry of WT Mtb into acid growth arrest, which identifies RegX3 as a novel regulator of acid growth arrest in Mtb. The enhanced growth phenotypes observed in low Pi conditions may be due to increased activation of the SenX3/RegX3 system, leading to increased phosphorylation of RegX3 and subsequent DNA binding. This activation likely decreases the amount of acid-induced ROS via upregulation of WhiB3 and subsequently preserves GAPDH activity, which is necessary for glycerol assimilation, and is further facilitated by increasing glycerol uptake via upregulation of *pe19* (Fig. 8). Further investigations are needed to determine the phosphorylation status of RegX3 and the impact of WhiB3 expression levels on GAPDH activity.

Our observation that PE19 participates in Δ*pstA1* growth on glycerol is supported by previous finding showing that the porin-like PE19-PPE51 complex is responsible for glycerol and glucose uptake in Mtb (45). Moreover, we showed that increased concentrations of glycerol, but not glucose, in media rescued the Mtb acid growth arrest especially in low Pi conditions. These data strengthen previous findings highlighting reduced glycerol uptake as an important factor in Mtb acid growth arrest (16, 19) and suggest that Mtb cannot use glucose to grow in acidic compartments during infection. As Δ*pstA1* and Δ*phoT* mutants phenocopy each other, our data also suggest that PhoT functions as the missing PstB subunit of Pst-1. In accordance, transposon mutants in Pst-1 encoding genes and *phoT* were previously shown to be functionally associated and required for Mtb fitness inside macrophages (46).

We and others have shown that Mtb faces acid stress inside macrophages (27, 47, 48), and previous work proposed Mtb experiences Pi limitation during infection (25, 30, 31, 49). It is reasonable to assume Mtb encounters both acid stress and Pi-limiting conditions inside phagosomes and thus has integrated systems to adapt to these stresses. The control of *whiB3* expression by RegX3 in response to both acidic pH and Pi-limiting conditions is reminiscent of similar mechanisms found in enterobacteria showing that Pi-specific TCS control the expression of acid-induced genes (50). Using a fluorescent protein under the control of *senX3* promoter, it was shown that the Mtb relative, *Mycobacterium marinum*, faces Pi-limiting conditions during infection in the zebrafish model (51). Such an experiment has yet to be performed to prove Pi-limitation in Mtb-containing phagosomes.

Mtb can access glycerol from lipid storage molecules such as triacylglycerols (TAGs) within macrophages (52). Furthermore, glycerol has been shown to contribute to susceptibility to TB in the context of type 2 diabetes in mice by serving as a nutrient for Mtb (53). Although glycerol assimilation is not required for Mtb virulence (54), we cannot exclude that Mtb utilizes glycerol, in addition to other carbon sources such as lipids, in specific environments such as in acidic and Pi-limiting compartments.

We show here that Mtb acid growth arrest can be manipulated by host-relevant cues such as Pi and glycerol levels as well as specific Mtb factors such as RegX3. Our study provides for the first time a direct role of Pi levels in regulating carbon utilization in Mtb and highlights the intricacy of Mtb regulation of acid stress response and nutrient utilization. Our data align with a model in which Mtb acid growth arrest is a genetically controlled program regulated by specific host-relevant cues. We hope this work will serve as a stepping stone toward a better understanding and targeting of new pathways necessary for Mtb adaptation to acid stress during infection.

## MATERIALS AND METHODS

### Bacterial strains and manipulation

Mycobacteria were grown in liquid culture in Middlebrook 7H9 medium supplemented with 0.2% glycerol, 0.05% tyloxapol, and ADN (0.5 g/L bovine serum albumin [BSA], 0.2% dextrose, and 0.085% NaCl), and grown on solid media of 7H10 agar supplemented with 0.5% glycerol and 10% Middlebrook OADC enrichment (Becton Dickinson). The Mtb mutant strains (H37Rv) Δ*pstA1,* Δ*phoT,* Δ*sdh1* and complemented mutants were constructed and characterized in our laboratory (see Mutant Generation and Complementation in Supplementary Material). The construction of the Mtb H37Rv Δ*pckA* mutant and complemented (Δ*pckA::pckA*) strains was performed in our laboratory as previously reported (17). The *regX3* overexpressing strain (WT::*regX3*) was constructed by introducing a second copy of *regX3* under the control of an anhydrotetracycline (ATc) inducible promoter into the *attL5* site of the genome of H37Rv. The following *Mtb* strains (Erdman background) used in this study were previously constructed and characterized in the laboratory of Anna Tischler: Δ*pstA1,* Δ*pstA1::pstA1,* Δ*regX3,* Δ*regX3::regX3,* Δ*pstA1*-Δ*regX3,* ∆*pstA1*-Δ*regX3::regX3,* Δ*pstA1*-Δ*pe19,* and Δ*pstA1*-Δ*pe19::pe19* (25, 55). The appropriate parental strain of *Mtb* (WT Erdman) was used as a control. Antibiotics were added to cultures when required at the following concentrations: hygromycin 50 µg/mL and kanamycin 25 µg/mL. Anhydrotetracycline (500 ng/mL) was added to induce *regX3* expression in WT::*regX3*.

### Bacterial cultures in various inorganic phosphate concentrations

In 7H9 medium, disodium and monopotassium phosphate provide around 25 mM Pi. To investigate the impact of Pi levels, an in-house Pi-free 7H9 base medium was prepared where both disodium and monopotassium phosphate were omitted. A supplement of fatty acid-free bovine serum albumin (5 g/L), NaCl (0.85 g/L), and glycerol (0.2, 0.5 or 1%) were added to the medium base. To ensure the medium is not devoid of potassium, potassium chloride (KCl) was added to a final concentration of 0.544 g/L. The Pi-free 7H9 base was supplemented with an appropriate buffering agent (MOPS at pH 7 or MES at pH 5) to maintain media buffering properties independent of Pi concentrations. Various concentrations of disodium phosphate were added to the Pi-free 7H9 medium base to evaluate the growth of *M. tuberculosis* at various Pi concentrations (Fig. S4). The Pi-free medium was used to prepare Low Pi (50 µM) and High Pi (25 mM) media at either pH 5 or pH 7 by adding appropriate amounts of disodium phosphate. Before inoculation, bacteria were grown in standard 7H9 media until mid-log phase and systematically washed three times with Pi-free 7H9 base media. For growth experiments, bacteria were inoculated at OD 0.05 and were grown at 37°C 5% CO_2_ in standing flasks. When used, oleic acid (OA) was supplemented every 2–3 days at a final concentration of 200 µM.

### Measurement of GAPDH activity and ROS production

Measurement of GAPDH activity and ROS in Mtb cultures depending on phosphate concentrations and pH were performed using the GAPDH activity assay kit (Sigma-Aldrich, MAK277-1KT) and the CellROX Green Reagent (Thermofisher scientific, C10444) as previously described (17) (see Supplementary Material and Methods).

### Gene expression analysis

After 5 days of incubation in specific media conditions, a 5M guanidine thiocyanate solution (Sigma-Aldrich, 5140) was added at a 1:1 vol to bacterial cultures followed by centrifugation for 10 min at 4°C. Bacterial pellets were re-suspended in Trizol and bead-beaten to release intracellular contents. RNA extraction was performed using the Zymo Research Clean and Concentrator kits (Zymo Research, R1015). The remaining genomic DNA was digested by DNAse treatment (Thermofisher Scientific, AM2239) before reverse transcription was performed (NEB, M0253L). Quantitative PCR was performed using a Roche Lightcycler 480 and Roche Mastermix reagents. Ct were quantified using the second derivative maximum and normalized to *M. tuberculosis sigA*. Primer and probe sequences are available upon request.

See Supplementary Material and Methods section for protocol details on the genome-wide mutagenesis screen, mutant generation, measurement of extracellular pH, and GAPDH detection by western blot.

## SUPPLEMENTAL MATERIAL

Data Set S1 mbio.02825-24-s0001.xlsx Tnseq screen results displaying the underrepresented and overrepresented hits at pH 5.5 vs pH 7.

Supplemental Figures mbio.02825-24-s0002.pdf Figures S1 to S12.

Supplemental Methods mbio.02825-24-s0003.docx Additional details on mutant generation and complementation, genome-wide mutagenesis screen, GAPDH activity assay, ROS and pH measurements, and Western blot.

ASM does not own the copyrights to Supplemental Material that may be linked to, or accessed through, an article. The authors have granted ASM a non-exclusive, world-wide license to publish the Supplemental Material files. Please contact the corresponding author directly for reuse.
