# Supplementary material for: An exacerbated phosphate starvation response triggers Mycobacterium tuberculosis glycerol utilization at acidic pH
Source: mBio. 2024 Nov 29;16(1):e02825-24. doi: 10.1128/mbio.02825-24 (PMC11708021; doi:10.1128/mbio.02825-24)
Supplement: Supplemental Figures — Figures S1 to S12. [file mbio.02825-24-s0002.pdf]

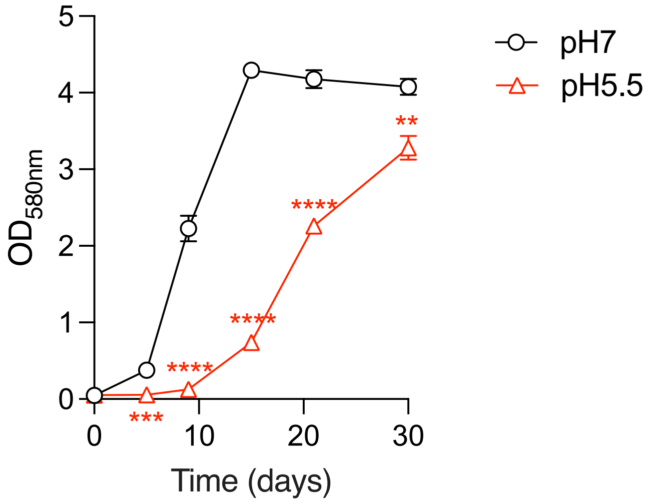

Figure S1. Growth of Mtb in standard 7H9 culture media at pH 7 and pH 5.5. Growth curve of Mtb H37Rv in complete 7H9 media (7H9 supplemented with 0.2%(v/v) glycerol, 0.05% tyloxapol, 5g/L BSA, 0.85g/L NaCl, 0.2% (w/v) dextrose) with pH adjusted to 7.0 or 5.5. Growth was monitored by measurement of optical density (OD_{590nm}). Data are the means and standard deviations of three independent experiments. Statistical significance was determined by unpaired t-test **:pval≤0.01, ***: p-val≤0.001, ****: p-val<0.0001

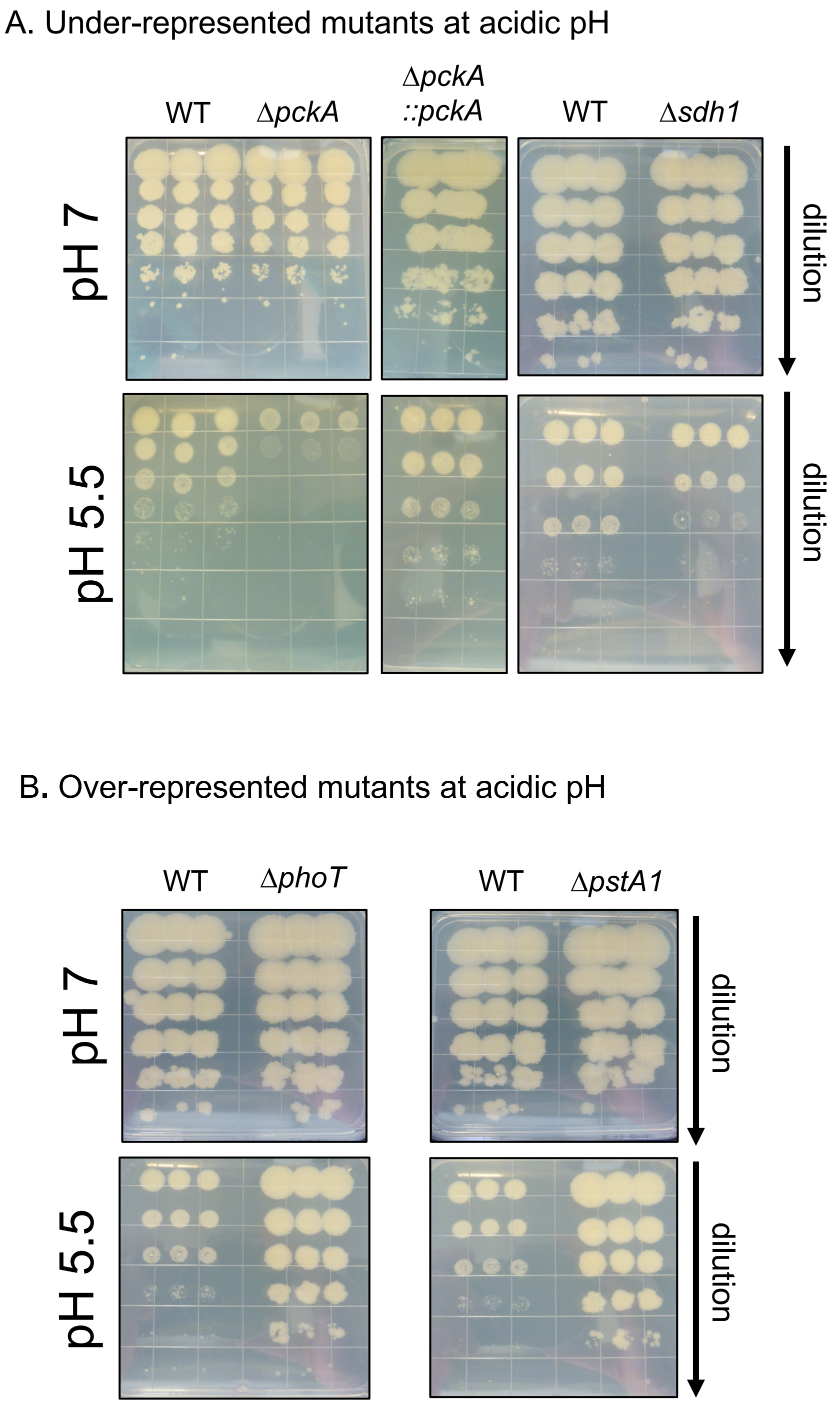

Figure S2. Validation of Tn-seq screen with genetic knock-out strains grown on 7H10 agar with pH adjusted to 7 or 5.5. (A) Validation of under-represented hits: Mtb wild type (WT), Mtb lacking *pckA* (Δ*pckA), pckA* complemented strain *(ΔpckA::pckA)* and Mtb lacking the succinate dehydrogenase complex 1 locus *(Δsdh1)* were serially diluted and 10μl drops placed onto complete 7H10 agar (7H10 agar supplemented with 0.5% glycerol and 10% OADC supplement) with adjusted pH of 7 or 5.5. (B) Validation of under-represented hits: Mtb wild type (WT), Mtb lacking *phoT* (Δ*phoT)* and *pstA1* (Δ*pstA1)* were serially diluted and 10μl drops placed onto complete 7H10 agar as described in A. pH7 plates were photographed after 21 days incubation, pH5.5 plates were photographed after 40 days. Images shown are representative of two independent experiments performed in triplicate.

A

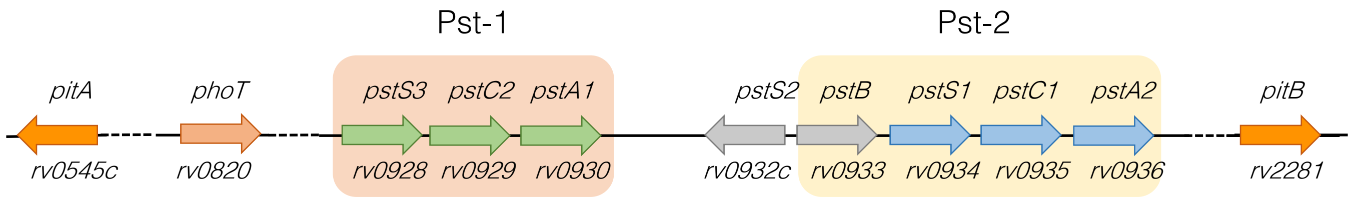

B

#Orf	Name	Description	log2FC	Adj. p-value
rv0545c	*pitA*	Probable low-affinity inorganic phosphate transporter integral membrane protein PitA	0.43	1
rv0820	***phoT***	**Probable phosphate-transport ATP-binding protein ABC transporter PhoT**	**3.88**	**0**
rv0928	***pstS3***	**Periplasmic phosphate-binding lipoprotein PstS3 (PBP-3) (PstS3) (PHOS1)**	**2.12**	**0.01826**
rv0929	***pstC2***	**Phosphate-transport integral membrane ABC transporter PstC2**	**3.49**	**0**
rv0930	***pstA1***	**Probable phosphate-transport integral membrane ABC transporter PstA1**	**2.94**	**0**
rv0932c	*pstS2*	Periplasmic phosphate-binding lipoprotein PstS2 (PBP-2) (PstS2)	-0.04	1
rv0933	*pstB*	Phosphate-transport ATP-binding protein ABC transporter PstB	-0.01	1
rv0934	*pstS1*	Periplasmic phosphate-binding lipoprotein PstS1 (PBP-1) (PstS1)	0.01	1
rv0935	*pstC1*	Phosphate-transport integral membrane ABC transporter PstC1	0.02	1
rv0936	*pstA2*	Phosphate-transport integral membrane ABC transporter PstA2	0.01	1
rv2281	*pitB*	Putative phosphate-transport permease PitB	0.69	1

Figure S3. Pst-1 and other inorganic phosphate transport systems in *M. tuberculosis*. (A) Genetic organisation of inorganic phosphate (Pi) transport systems in Mtb. (B) Tn-seq data of Pi transport genes and their requirement for Mtb to grow at pH 5.5 compared to pH 7. For each gene, the ratio of normalized sequence reads per insertion site (pH 5.5/pH 7) was determined (log_2 fold change: Log2FC). Data are from three biological replicate experiments. Mutants with insertions in genes that were significantly overrepresented at pH 5.5 are listed in bold.

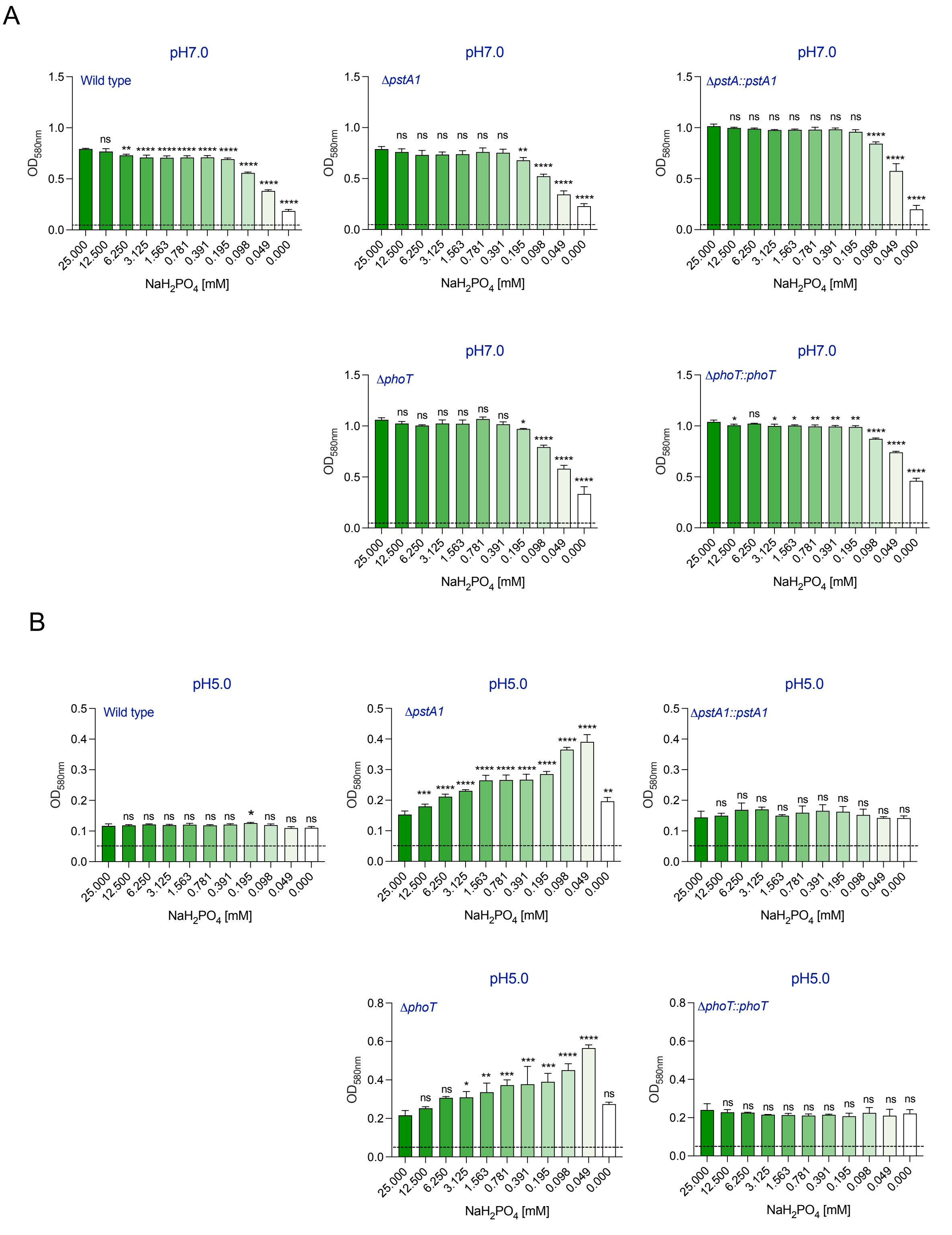

Figure S4. Growth of Mtb lacking the genes *pstA1* and *phoT* in media at pH 5 and pH 7 in the presence of a gradient of Pi concentrations. Mtb wild type (WT), mutants (Δ*pstA1*, Δ*phoT*) and complemented strains (Δ*pstA1::pstA1(ΔpstA1-comp),* Δ*phoT::phoT(ΔphoT-comp*) were grown in 7H9 containing glycerol-0.2% and glucose-0.2% as main carbon sources (prepared in-house) with a range of inorganic phosphate concentrations (disodium phosphate) and adjusted to pH 7 (A) or 5 (B). Growth was measured by optical density OD$_{590nm}$ on day 15. Dashed line indicates OD$_{590nm}$ at day 0 (0.05). Statistical significance compared to 25mM Pi was determined by ordinary one-way ANOVA with Dunnett multiple comparisons test. *:p-val≤0.05, **:pval≤0.01, ***: p-val≤0.001, ****: p-val<0.0001, ns: not significant

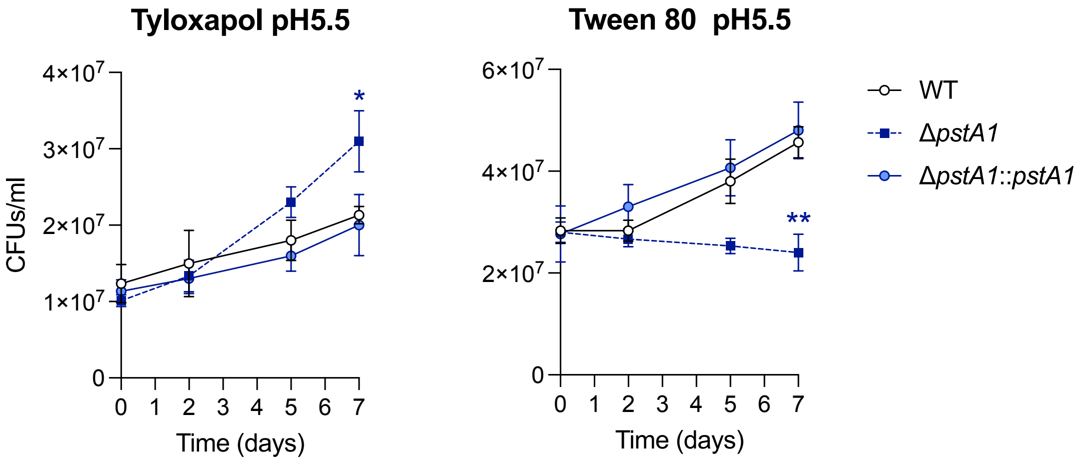

Figure S5. Mtb lacking *pstA1* is sensitive to the detergent Tween80 in acidic media. Survival of wild type (WT), *pstA1* knock-out (Δ*pstA1)* and complemented mutant (Δ*pstA1::pstA1)* in 7H9 pH 5.5 media supplemented with either tyloxapol (0.05%) or Tween 80 (0.1%) . Data is representative of two independent experiments, performed in triplicate. Statistical significance at day was determined by one-way ANOVA and Tukey multiple comparisons test. *:pval≤0.05, **:pval≤0.01

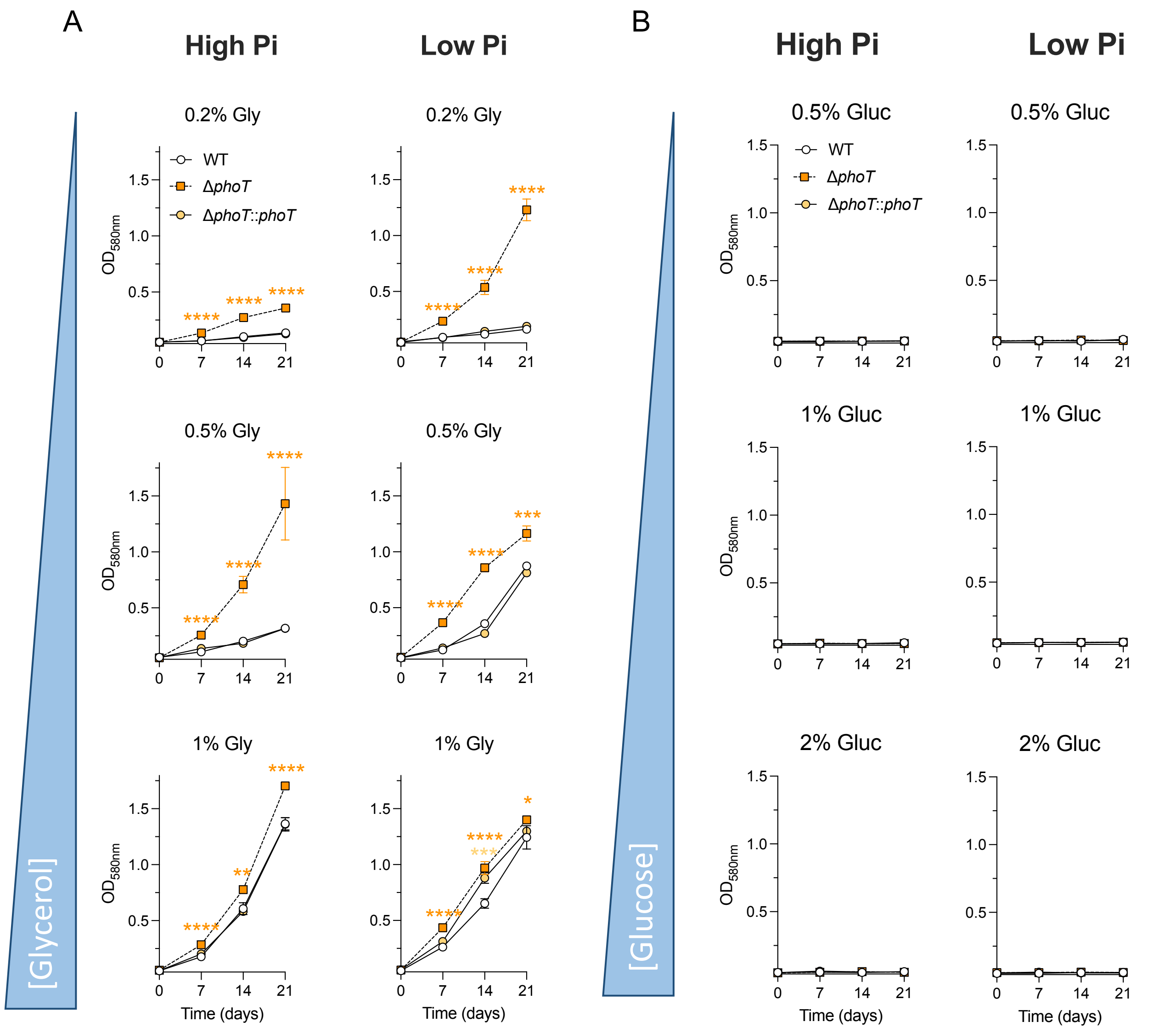

Figure S6. Mtb lacking *phoT* displays an increased ability to utilize glycerol but not glucose to grow at acidic pH. Growth of wild type Mtb (WT), Δ*phoT* and complemented mutant (Δ*phoT*::Δ*phoT*) in 7H9 at pH 5 with High (25mM) or Low (50µM) inorganic phosphate (Pi). Growth was determined for each strain in a gradient of glycerol (Gly) (A) or glucose (Gluc) (B) concentrations (% v/v) that serves as main carbon source. Growth was monitored by measurement of optical density (OD_{590nm}). Data are the means and standard deviations of three independent experiments. Statistical significance was determined by ordinary one-way ANOVA with Tukey multiple comparisons test. *:pval≤0.05, **:pval≤0.01, ***: p-val≤0.001, ****: p-val<0.0001

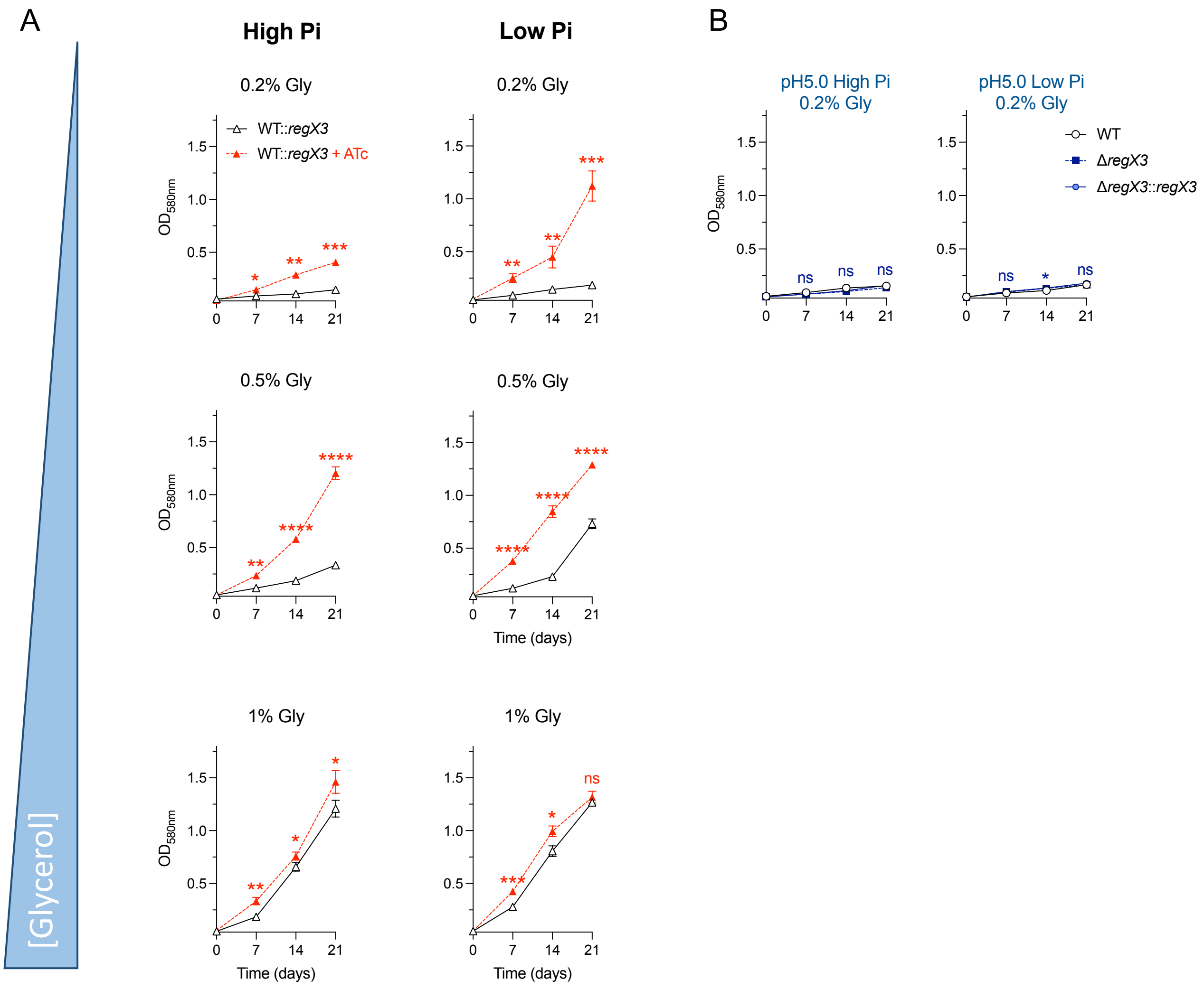

Figure S7. Mtb overexpressing RegX3 displays an increased ability to utilize glycerol to grow at acidic pH. (A) Growth of wild type Mtb (WT) containing a second copy of *regX3* under the control of an Anhydrotetracycline (ATc) inducible promoter (WT::*regX3*) in 7H9 media at pH 5 with high inorganic phosphate (Pi) (25mM) or low Pi (50µM). ATc [500ng/mL] was added to cultures to induce *regX3* expression. Growth was determined for each strain in a gradient of glycerol (Gly) concentrations (% v/v) that serves as main carbon source. (B) Growth of WT Mtb , *regX3* knock out mutant (Δ*regX3*) and complemented mutant Δ*regX3::regX3* in 7H9 0.2% glycerol media at pH 5 with high Pi or low Pi. Growth was monitored by measurement of optical density (OD_{590nm}). Data are the means and standard deviations of three independent experiments. Statistical significance for A was determined by unpaired t-test. Statistical significance for B was determined by one-way ANOVA with Tukey multiple comparisons test. *:pval≤0.05, **:pval≤0.01, ***: p-val≤0.001, ****: p-val<0.0001, ns: not significant

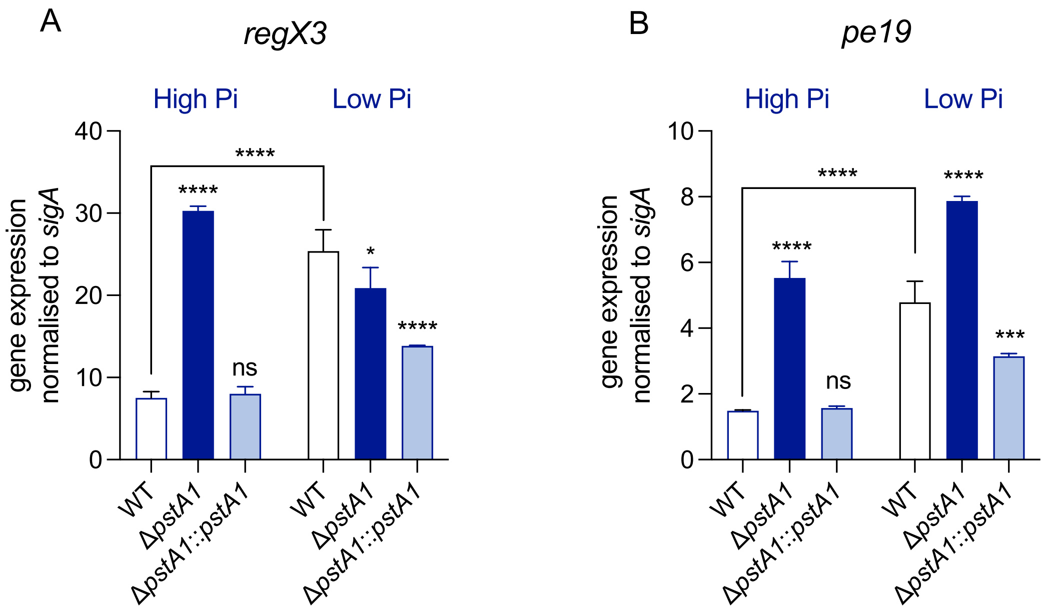

Figure S8. Expression pattern of *regX3* and *regX3*-regulated genes *pe19* and *whiB3* in High or Low inorganic phosphate (Pi) conditions at pH 7. Gene expression analysis of *regX3* (A) and *pe19* (B) in wild type (WT), *pstA1* knock-out mutant (Δ*pstA1)* and complemented mutant (Δ*pstA1::pstA1)* in 7H9-0.2%glycerol media at pH 7 with high Pi (25mM) and low Pi (50µM). Data are the means and standard deviations of three independent experiments. Statistical significance was determined by one-way ANOVA and Tukey multiple comparisons test. *:pval≤0.05, ***: p-val≤0.001, ****: p-val<0.0001

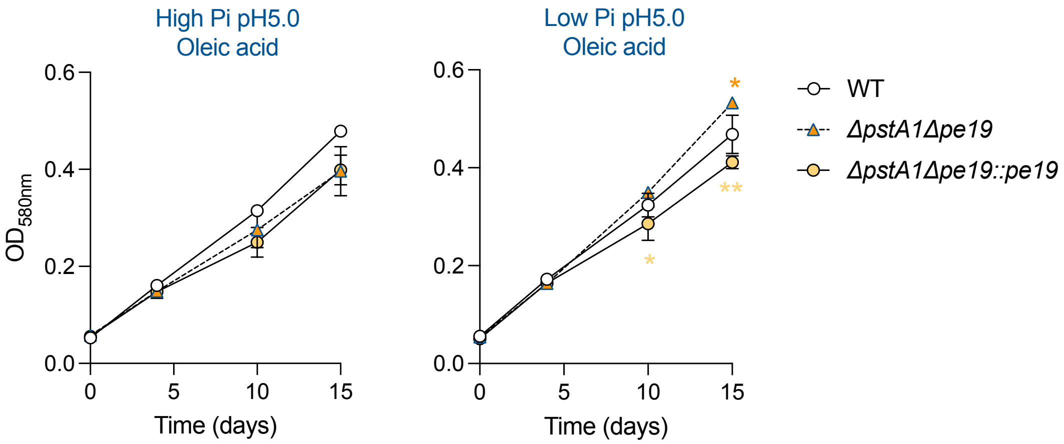

Figure S9. PE19 is not required for optimal growth of Δ*pstA1* at pH 5 with oleic acid as a main carbon source. Growth curve experiments of wild type (WT), *pstA1* and *pe19* double knock-out (Δ*pstA1*Δ*pe19*) and double knock-out complemented strain *(ΔpstA1Δpe19::pe19)* in 7H9 at pH 5 with high Pi (25mM) and low Pi (50μM) and with oleic acid as the main carbon source (200 μM oleic acid replenished every 2-3 days). Data are the means and standard deviations of three independent experiments. Statistical significance at each timepoint was determined by one-way ANOVA and Tukey multiple comparisons test. *:pval≤0.05, **:pval≤0.01.

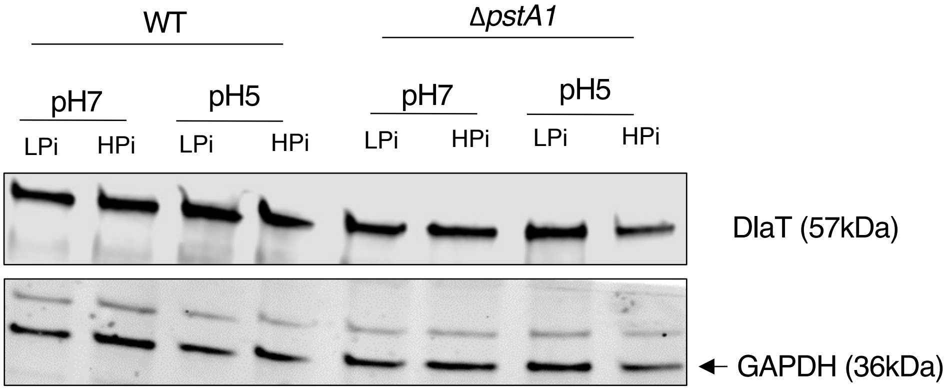

Figure S10. Loss of *pstA1* in Mtb does not affect GAPDH protein levels. Western blotting analysis of wild type (WT) and *pstA1* knock-out (Δ*pstA1)* in pH 7 and pH 5 media with high (Hpi; 25mM) or low Pi (Lpi; 50μM). Representative blot from two independent experiments. 50μg of total protein were probed for GAPDH detection. DlaT was used as a loading control.

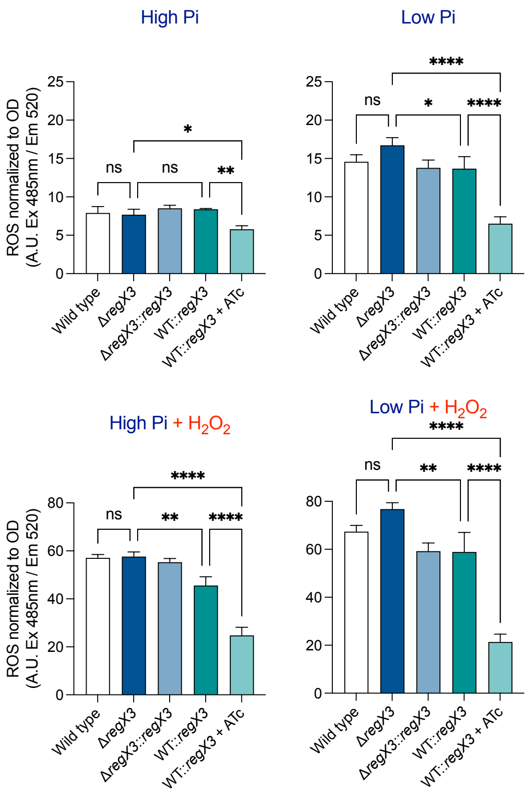

Figure S11. Loss of RegX3 does not affect reactive oxygen species (ROS) levels in conditions of limited phosphate and oxidative stress in acidic conditions. (A) Measurements of intracellular ROS in *M. tuberculosis* strains cultured for 5 days in 7H9-0.2%glycerol at pH 5 with high (25mM) or low (50µM) inorganic phosphate (Pi). (B) Same experiment as in (A) with the addition of hydrogen peroxide H_2O_2 (1mM) to the cultures to induce oxidative stress during ROS measurements. Levels of ROS in (A) and (B) were measured by fluorescence measurement of the Cellrox Green probe (5 µM) 2 hours after its addition. Data are the means and standard deviations and representative of two experiments performed in triplicate. Data in C are the means and standard deviations of three biological replicates. Statistical significance was determined by ordinary one-way ANOVA with Tukey multiple comparisons test. *:p-val≤0.05, **:pval≤0.01, ***: p-val≤0.001, ****: p-val<0.0001

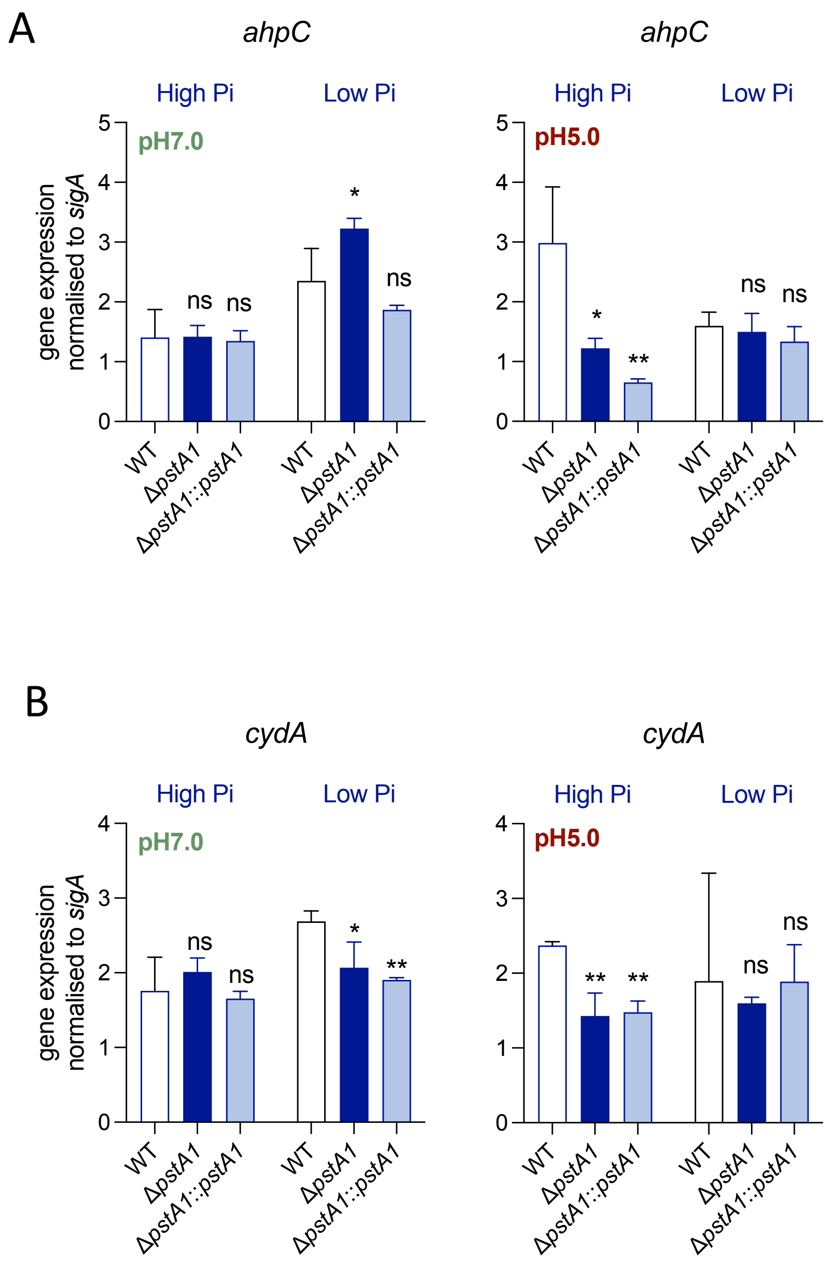

Figure S12. Mtb lacking *pstA1* does not display a substantial increase in *ahpC* and *cydA* expression in our growth conditions. Gene expression analysis of *ahpC* (A) and *cydA* (B) in wild type (WT), *pstA1* knock-out (Δ*pstA1)* and complemented mutant (Δ*pstA1::pstA1)* in pH 7 and pH 5 media with high (25mM) or low Pi (50µM). Data are the mean and standard deviation of three independent experiments. Statistical significance was determined by one-way ANOVA and Dunnett multiple comparisons test. *:pval≤0.05, **:pval≤0.01, ns: not significant
