# Supplementary material for: An exacerbated phosphate starvation response triggers Mycobacterium tuberculosis glycerol utilization at acidic pH
Source: mBio. 2024 Nov 29;16(1):e02825-24. doi: 10.1128/mbio.02825-24 (PMC11708021; doi:10.1128/mbio.02825-24)
Supplement: Supplemental Methods — Additional details on mutant generation and complementation, genome-wide mutagenesis screen, GAPDH activity assay, ROS and pH measurements, and Western blot. [file mbio.02825-24-s0003.docx]

**Supplemental material and methods**

**Mutant generation and complementation.**

Knockout mutants Δ*pstA1*, Δ*phoT* and Δ*sdh1* were generated in *M. tuberculosis* H37Rv by recombineering as previously described (1). The knockout cassettes were synthesized by Genescript and consisted in an hygromycin cassette flanked with 500 bp upstream and 500 bp downstream regions of the genes to be replaced. In the case of Δ*sdh1*, the three genes *rv0247c*, *rv0248c* and *rv0249c* were deleted. Some nucleotides were left at the start and at the end of the replaced genes (Start nucleotide/End nucleotide) as follow Δ*pstA1* (8nt/26nt), Δ*phoT* (6nt/7nt) and Δ*sdh1* (7nt at the start of *rv0249c* and 20nt at the end of *rv0247c*). Recombinants were selected on 7H10 agar plates containing hygromycin. Complementation of Δ*pstA1* and Δ*phoT* was achieved by expressing the genes *pstA1* (Δ*pstA1::pstA1*) and *phoT (*Δ*phoT::phoT*) under their native promoter. *pstA1* native promoter was selected as a 250bp DNA fragment upstream the ATG start codon of the gene *pstS3* (*rv0928*). *phoT* native promoter was selected as a 250bp DNA fragment upstream the ATG start codon of *phoT*. The plasmids used for complementation were generated using Gateway cloning technology (Life Technologies). Plasmids contained a kanamycin resistance cassette and were integrative. The strains (H37Rv background) WT, Δ*pstA1,* Δ*pstA1::pstA1,* Δ*phoT* and Δ*phoT::phoT* were used for the experiments displayed in figures 2 & 3 and the supplemental figures S2, S4 and S5. Our strains (H37Rv background) displayed the same phenotype than the corresponding strains (Erdman background) gifted by the laboratory of Anna Tischler.

**Genome wide mutagenesis screen**

An *M. tuberculosis* H37Rv saturated transposon library was constructed by *himar1* mutagenesis as previously described (2). The saturated transposon mutant library was cultured on solid media (Middlebrook 7H10) buffered to either pH 7 or pH 5.5 and containing glycerol (Gly = 0.5% ~ 54 mM) and glucose (Glu = 0.2% ~ 11mM) as main carbon sources and traces of oleic acid (OA ~ 200 µM). Colonies were harvested by scraping into TE buffer after 3 weeks (pH 7) or 6 weeks (pH 5.5) of incubation at 37ºC. The 3-week growth delay observed for the libraries grown on acidified agar plates is consistent with the extended lag phase observed for Mtb grown in liquid media (Fig. S1) (3, 4). Genomic DNA was extracted from the transposon libraries, and the library mutant composition was determined by sequencing amplicons of the transposon-genome junctions as described previously (5, 6). Mapping and quantification of transposon insertions was done as described previously (2, 5). The TRANSIT Tn-seq analysis tool (7) was used to identify transposon mutants that were under- or over-represented in acidic conditions (log_2_ fold change, greater than 2 or less than −2) with statistical significance after correction for multiple comparisons (*q* < 0.05).

**GAPDH activity assay**

Measurement of GAPDH activity in Mtb lysates cultured in different phosphate concentrations and pH were performed using the GAPDH activity assay kit (Sigma-Aldrich, MAK277-1KT) as previously described (8). Briefly, bacteria were grown in standard 7H9 media until mid-log phase and then wash 3 times with Pi-free 7H9 media and inoculated at final OD of 0.5 in 25 mL of Low or High Pi media at pH 7 or pH 5. After 5 days incubation at 37 °C, ODs were measured, and bacterial volumes collected were adjusted to pellet a similar biomass. The pellets were then resuspended in GAPDH activity assay buffer before mechanical lysis with 0.1 mm zirconia beads in a Precellys tissue homogenizer for 20 s (5,000 rpm) twice under continuous cooling. In total, 50 μL lysates were tested in each condition. Lysate protein content were determined using Qubit protein assay kit (Thermofisher scientific), and results were normalized per mg of proteins (NADH/protein [nM/mg]). GAPDH activity (milliunits/mg protein) was determined using the calculation from the GAPDH activity assay kit (GAPDH activity (milliunit/mL) = NADH (nmol)/(Time (min) × Volume lysate (mL)) and divided by the quantity of proteins from each lysate (mg).

**ROS measurements**

Bacteria were grown in standard 7H9 media until mid-log phase and then washed 3 times with Pi-free 7H9 media and inoculated at final OD of 0.2 in 20 mL of Low or High Pi media at pH 5. After 5 days incubation at 37 °C, bacteria were spun down and resuspended in 1 mL of the same medium. OD were adjusted to OD = 0.5 with corresponding media and 200 μL bacteria were added to a 96-well plate (black plate/clear bottom). OD 600 nm was recorded for future normalization. To measure ROS generation, CellROX Green Reagent (Thermofisher scientific, C10444) was added to the wells at a final concentration of 5 μM as previously described (8). Each condition was recorded in triplicate wells, and a mean was calculated. Recording of fluorescence was done at 37 °C for 2 h using a plate reader (excitation 485 nm/emission 520 nm). Fluorescence measurements were normalized to OD (Fluo/OD) and expressed as arbitrary units (A.U.) excitation 485 nm/ emission 520 nm.

**Measure of extracellular pH**

To measure the pH of cultures (extracellular pH; pHEX), a pH indicator dye, chlorophenol red, was used as previously described (8). Samples of *M. tuberculosis* cultures (250 μL) were mixed with 0.004% chlorophenol red, and cells were pelleted by centrifugation. A total of 200 μL supernatant was transferred to wells in 96-well plate. The absorbance at 430 and 590 nm was measured, and the 430/590 nm ratio was calculated and plotted onto a calibration curve to determine the pH. For each batch of chlorophenol red, a calibration curve was generated using a set of media samples with adjusted pH values from 6.6 to 4.2.

**Western Blot**

After 5 days incubation in the tested media, cultures were pelleted and lysed by bead-beating in phosphate buffered saline containing a protease inhibitor cocktail (Roche). Lysates were sterilized by passing through 0.22 μm Spin-X filters (Costar) and protein content was quantified using Qubit (Thermofisher scientific, A50668). Protein lysates were denatured in Laemmli buffer containing 2-Mercaptoethanol at 100ºC for 2 min. 50 μg total protein were separated by SDS–PAGE and transferred to nitrocellulose membranes for probing with rabbit antisera against Mtb GAPDH and dihydrolipoamide acyltransferase (DlaT). Goat anti-rabbit IgG (LI-COR Biosciences) was used as the secondary antibody and blots were developed using the Odyssey Infrared Imaging System (LICOR Biosciences).

**References**

1. Murphy KC, Papavinasasundaram K, Sassetti CM. 2015. Mycobacterial Recombineering, p. 177–199. *In* Parish, T, Roberts, DM (eds.), Mycobacteria Protocols. Springer, New York, NY.

2. Xu W, DeJesus MA, Rücker N, Engelhart CA, Wright MG, Healy C, Lin K, Wang R, Park SW, Ioerger TR, Schnappinger D, Ehrt S. 2017. Chemical Genetic Interaction Profiling Reveals Determinants of Intrinsic Antibiotic Resistance in Mycobacterium tuberculosis. Antimicrobial agents and chemotherapy 61:e01334-17.

3. Baker JJ, Abramovitch RB. 2018. Genetic and metabolic regulation of Mycobacterium tuberculosis acid growth arrest. Scientific Reports 8:4168.

4. Baker JJ, Johnson BK, Abramovitch RB. 2014. Slow growth of Mycobacterium tuberculosis at acidic pH is regulated by phoPR and host-associated carbon sources. Molecular microbiology https://doi.org/10.1111/mmi.12688.

5. DeJesus MA, Gerrick ER, Xu W, Park SW, Long JE, Boutte CC, Rubin EJ, Schnappinger D, Ehrt S, Fortune SM, Sassetti CM, Ioerger TR. 2017. Comprehensive Essentiality Analysis of the Mycobacterium tuberculosis Genome via Saturating Transposon Mutagenesis. mBio 8:10.1128/mbio.02133-16.

6. Long JE, DeJesus M, Ward D, Baker RE, Ioerger T, Sassetti CM. 2015. Identifying Essential Genes in Mycobacterium tuberculosis by Global Phenotypic Profiling, p. 79–95. *In* Lu, LJ (ed.), Gene Essentiality: Methods and Protocols. Springer, New York, NY.

7. DeJesus MA, Ambadipudi C, Baker R, Sassetti C, Ioerger TR. 2015. TRANSIT--A Software Tool for Himar1 TnSeq Analysis. PLoS Comput Biol 11:e1004401.

8. Gouzy A, Healy C, Black KA, Rhee KY, Ehrt S. 2021. Growth of Mycobacterium tuberculosis at acidic pH depends on lipid assimilation and is accompanied by reduced GAPDH activity. Proc Natl Acad Sci U S A 118:e2024571118.
